# Comparative Analysis of Global Proteome and Lysine Acetylome Between Naive CD4^+^ T Cells and CD4^+^ T Follicular Helper Cells

**DOI:** 10.3389/fimmu.2021.643441

**Published:** 2021-03-25

**Authors:** Ming Zhao, Sujie Jia, Xiaofei Gao, Hong Qiu, Ruifang Wu, Haijing Wu, Qianjin Lu

**Affiliations:** ^1^Department of Dermatology, Hunan Key Laboratory of Medical Epigenomics, The Second Xiangya Hospital of Central South University, Changsha, China; ^2^Research Unit of Key Technologies of Diagnosis and Treatment for Immune-Related Skin Diseases, Chinese Academy of Medical Sciences, Changsha, China; ^3^Department of Pharmaceutics, The Third Xiangya Hospital of Central South University, Changsha, China

**Keywords:** lysine acetylation, histone acetyltransferases, histone deacetylases, follicular helper T cell, proteome, acetylome

## Abstract

As a subgroup of CD4^+^ T helper cells, follicular helper T (Tfh) cells provide help to germinal center B cells and mediate the development of long-lived humoral immunity. Dysregulation of Tfh cells is associated with several major autoimmune diseases. Although recent studies showed that Tfh cell differentiation is controlled by the transcription factor Bcl6, cytokines, and cell-cell signals, limited information is available on the proteome and post-translational modifications (PTMs) of proteins in human Tfh cells. In the present study, we investigated quantitative proteome and acetylome in human naive CD4^+^ T cells and *in vitro* induced Tfh (iTfh) cells using the tandem mass tag (TMT) labeling technique, antibody-based affinity enrichment, and high-resolution liquid chromatography-mass spectrometry (LC-MS)/mass spectrometry (MS) analysis. In total, we identified 802 upregulated proteins and 598 downregulated proteins at the threshold of 1.5-fold in iTfh cells compared to naive CD4^+^ T cells. With the aid of intensive bioinformatics, the biological process, the cellular compartment, the molecular function, Kyoto Encyclopedia of Genes and Genomes (KEGG) pathway, and protein–protein interaction of these differentially expressed proteins were revealed. Moreover, the acetylome data showed that 22 lysine (K) acetylated proteins are upregulated and 26 K acetylated proteins are downregulated in iTfh cells compared to the naive CD4^+^ T cells, among which 11 differentially acetylated K residues in core histones were identified, indicating that protein acetylation and epigenetic mechanism are involved in regulating Tfh cell differentiation. The study provides some important clues for investigating T cell activation and Tfh cell differentiation.

## Introduction

Follicular helper T (Tfh) cells have emerged as a separate CD4^+^ T helper subset specialized in assisting B cells in recent times. Tfh cells play a key part in humoral immunity, involving the generation of long-lived and high-affinity plasma cells and memory cells, which are essential for long-term defense for infections (1, 2). Recent studies suggested that Tfh cell differentiation and function are essential in the control of virus infections (2–5). Moreover, Tfh cell expansion has been observed in patients with autoimmune diseases, such as systemic lupus erythematosus (SLE) (6–8) and rheumatoid arthritis (RA) (9), and in several mouse models of autoimmunity (10, 11) and was shown to play a pathogenic role in the pathogenesis of disease in some models (12, 13). Therefore, elucidating the underlying molecular biological mechanisms of Tfh cell differentiation and function is of great importance for identifying the novel targets of therapy for autoimmune diseases.

Proteins perform many biological functions within organisms, including DNA replication, catalyzing metabolic reactions, response to stimuli, molecule transportation, and so on. Proteomic changes consisting of signaling and organization in the cell nucleus are significant in both the initial and the late stages, leading to Th cells differentiation (14, 15). Moreover, post-translational modifications (PTMs) can influence inflammation by targeting natural sensors and downstream signal molecules (such as enzymes, adaptors, receptors, and transcriptional factors) (16). Lysine acetylation (Kac) residues is one type of PTMs and plays a key role in cellular metabolism, chromatin remodeling, cytoplasmic and nuclear transport, and mitosis (17). Kac residues can be regulated by acetyltransferases and deacetylases. Acetylation of protein plays a vital role in the regulation of inflammasome-dependent innate immune responses, toll-like receptors, and retinoic-acid-inducible gene I (RIG-I)-like receptors (18, 19).

The differentiation process of the Th cells leads to the heritable and specific gene expression profiles without changing the DNA base sequence. Therefore, epigenetics play a vital role in the determination of the fate of Th cell specification (20, 21). Kac is one of the most important epigenetic mechanisms and is closely related to gene transcriptional activation and inhibition. Kac residues in core histones can neutralize the positive charge of histone side chains and enhance the interaction between histones and negatively charged DNA, which promotes a more relaxed chromatin structure so that chromatin would allow transcriptional activation. Reversely, histone deacetylation leads to a more tight interaction between negatively charged DNA and positively charged histones, which are generally associated with chromatin condensation and are not conducive to transcription (22). Histone acetylation is important in regulating the differentiation and functions of Th cell subsets (23, 24).

**Figure 1:**
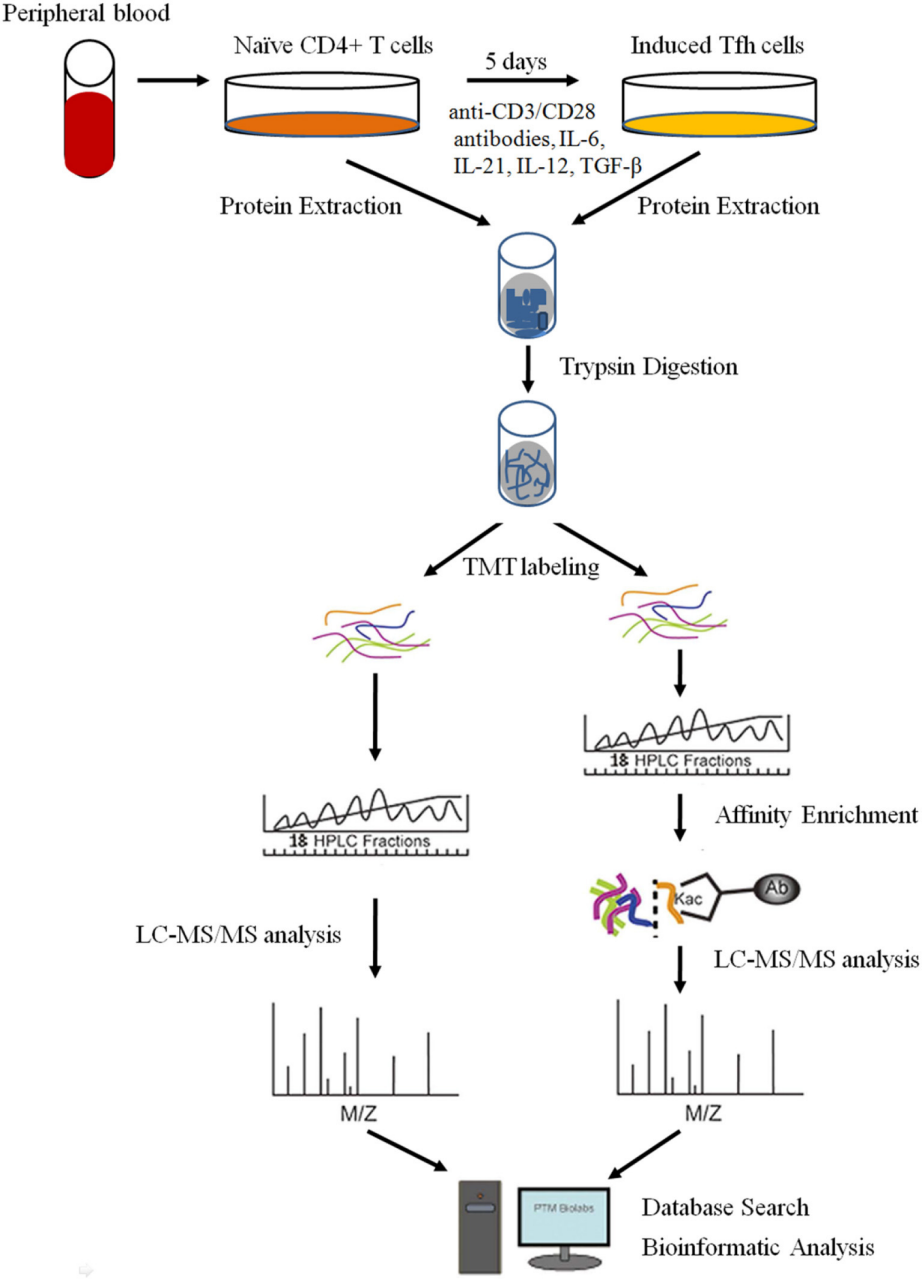
The workflow for the quantitative analysis of global proteome and lysine (K) acetylome in naive CD4^+^ T cells and induced Tfh (iTfh) cells.

Despite the knowledge that some important proteins, such as IL-21, BCL6, CD40L, and Blimp-1, are critical for the differentiation and function of Tfh cells (2, 12), the underlying mechanism of PTMs and proteome networks remain largely unknown. In the present study, we explored the global proteome and lysine (K) acetylome of naive CD4^+^ T cells and intensively induced Tfh (iTfh) cells *in vitro* with TMT labeling, high-efficiency acetylation enrichment, and high-resolution liquid chromatography-mass spectrometry (LC-MS)/mass spectrometry (MS) analysis (Figure 1). We identified 5,148 proteins and 281 Kac sites successfully. With progressive bioinformatics methods, we aimed at exploring the potential molecular mechanisms underlying T cell activation and Tfh cell differentiation, which may promote the understanding of the pathogenesis of some autoimmune diseases.

## Experimental Procedures

### Isolation of Naive CD4^+^ T Cells and Induced Differentiation of Tfh Cells *in vitro*

This study was approved by the Ethics Committee of the Second Xiangya Hospital of Central South University. Peripheral blood mononuclear cells (PBMCs) were separated from the peripheral blood of healthy participants by density gradient centrifugation (GE Healthcare, Boston, MA, USA). Healthy participants were recruited from the medical staff at the Second Xiangya Hospital. CD4^+^CD45RA^+^ naïve T cells were isolated by negative selection using Naive CD4^+^ T Cell Isolation Kit II (Miltenyi Biotec, Bergisch Gladbach, Germany) according to the instructions of the manufacturer. The purity of the enriched subset was validated by flow cytometry with anti-CD4 and CD45RA antibodies (BD Biosciences, San Jose, CA, USA) and was generally higher than 90%. Tfh cell differentiation: purified CD4^+^CD45RA^+^ naïve T cells were seeded in anti-CD3 antibody (5 μg/ml, Calbiochem, San Diego, CA, USA) pre-coated 24-well plates (5 × 10^5^ cells/ml) in the presence of anti-CD28 antibody (2 μg/ml, Calbiochem), IL-6 (20 ng/ml, Peprotech, Cranbury, NJ, USA), IL-21 (20 ng/ml, Peprotech), IL-12 (10 ng/ml, Peprotech), and TGF-β (5 ng/ml, Peprotech) for 5 days (7, 25). The medium was refreshed on Day 3. iTfh cells were identified by flow cytometry with anti-CXCR5 and PD-1 antibodies (BD Biosciences). The cells were harvested for proteome and acetylome analyses.

### Proteome TMT Labeling and LC-MS/MS Analysis

#### Protein Extraction

Cells were transferred to a 5-ml centrifuge tube and sonicated three times on ice using a high-intensity ultrasonic processor in lysis buffer [8 M urea, 2 mM Ethylene Diamine Tetraacetic Acid (EDTA), 10 mM dithiothreitol (DTT), and 1% Protease Inhibitor Cocktail]. The remaining debris was removed by centrifugation at 20,000 g at 4°C for 10 min. The protein was precipitated with 15% cold trichloroacetic acid (TCA) for 4 h at −20°C. After centrifugation at 4°C for 3 min, the supernatant was discarded. The remaining precipitate was washed three times with cold acetone. The protein was redissolved in the buffer [8 M urea, 100 mM tetraethyl-ammonium bromide (TEAB), pH 8.0], and the protein concentration in the supernatant was determined with a 2-D Quant kit (Cytiva, Marlborough, MA, USA) according to the instructions of the manufacturer.

#### Trypsin Digestion

For digestion, the protein solution was reduced with 10 mM DTT for 1 h at 37°C and alkylated with 20 mM iodoacetamide (IAA) for 45 min at room temperature in darkness. For trypsin digestion, the protein sample was diluted by adding 200 mM TEAB to a urea concentration of <2 M. Finally, trypsin (Promega, Madison, WI, USA) was added at 1:50 trypsin-to-protein mass ratio for the first digestion overnight and to 1:100 trypsin-to-protein mass ratio for a second 4 h-digestion. Approximately, 100 μg protein for each sample was digested with trypsin for the following experiments.

#### TMT Labeling

After trypsin digestion, the peptide was desalted by Strata X C18 SPE column (Phenomenex, Torrance, CA, USA) and vacuum-dried. The peptide was reconstituted in 1 M TEAB and processed according to the protocol of the manufacturer for a TMTsixplex Isobaric Mass Tagging kit (ThermoFisher Scientific, Massachusetts, USA). Briefly, one unit of TMT reagent (defined as the amount of reagent required to label 100 μg of protein) was thawed and reconstituted in 24 μl acetonitrile (ACN). The peptide mixtures were then incubated for 2 h at room temperature and were pooled, desalted, and dried by vacuum centrifugation.

#### HPLC Fractionation

The sample was then fractionated into fractions by high pH reverse-phase HPLC using an Agilent 300Extend C18 column. Briefly, peptides were first separated with a gradient of 2–60% ACN in 10 mM ammonium bicarbonate pH 10 over 80 min into 80 fractions. Then, the peptides were combined into 18 fractions and dried by vacuum centrifuging.

#### Affinity Enrichment (for Acetylome Analysis)

To enrich Kac peptides, tryptic peptides dissolved in NETN buffer (100 mM NaCl, 1 mM EDTA, 50 mM Tris-HCl, 0.5% NP-40, pH 8.0) were incubated with pre-washed antibody beads at 4°C overnight with gentle shaking. The beads were washed four times with NETN buffer and two times with ddH_2_O. The bound peptides were eluted from the beads with 0.1% trifluoroacetic acid (TFA). The eluted fractions were combined and vacuum-dried. The resulting peptides were cleaned with C18 ZipTips (Merck Millipore, Darmstadt, Germany) according to the instructions of the manufacturer, followed by LC-MS/MS analysis.

#### LC-MS/MS Analysis

Peptides were dissolved in 0.1% formic acid (FA) and directly loaded onto a reversed-phase pre-column (Acclaim PepMap 100, ThermoFisher Scientific). Peptide separation was performed using a reversed-phase analytical column (Acclaim PepMap RSLC, ThermoFisher Scientific). The gradient was comprised of an increase from 5 to 25% solvent B (0.1% FA in 98% can) over 26 min, from 25 to 40% in 8 min and climbing to 80% in 3 min and then holding at 80% for the last 3 min, all at a constant flow rate of 400 nl/min on an EASY-nLC 1000 UPLC system. These resulting peptides were analyzed by Q ExactiveTM hybrid quadrupole-Orbitrap mass spectrometer (ThermoFisher Scientific).

The peptides were subjected to NSI source followed by tandem mass spectrometry (MS/MS) in Q Exactive™ (ThermoFisher Scientific) coupled online to the ultra-performance liquid chromatography (UPLC). Intact peptides were detected in the Orbitrap at a resolution of 70,000. Peptides were selected for MS/MS using NCE setting as 28 ion fragments were detected in the Orbitrap at a resolution of 17,500. A data-dependent procedure that alternated between one MS scan and 20 MS/MS scans was applied for the top 20 precursor ions above a threshold ion count of 1E4 in the MS survey scan with 30.0 s dynamic exclusion. The electrospray voltage applied was 2.0 kV. Automatic gain control (AGC) was used to prevent overfilling of the ion trap; 5E4 ions were accumulated for the generation of MS/MS spectra. For MS scans, the m/z scan ranged from 350 to 1,800, and fixed first mass was set to 100 m/z.

### Database Search

#### Proteome

The resulting MS/MS data were processed using the Mascot search engine (v.2.3.0). Tandem mass spectra were searched against the SwissProt Human database. Trypsin/P was specified as a cleavage enzyme allowing up to two missing cleavages. The mass error was set to 10 ppm for precursor ions and to 0.02 Da for fragment ions. Carbamidomethyl on Cys was specified as a fixed modification, and oxidation on Met was specified as variable modifications. For the protein quantification method, TMTsixplex was selected by the Mascot engine. False discovery rate (FDR) was adjusted to <1%, and the peptide ion score was set ≥20.

#### Acetylome

The resulting MS/MS data were processed using MaxQuant with an integrated Andromeda search engine (v.1.4.1.2). Tandem mass spectra were searched against the Swissprot Human database concatenated with reverse decoy database. Trypsin/P was specified as a cleavage enzyme, allowing up to four missing cleavages, five modifications per peptide, and five charges. The mass error was set to 10 ppm for precursor ions and to 0.02 Da for fragment ions. Carbamidomethylation on Cys was specified as a fixed modification, and oxidation on Met, acetylation on Lys, and acetylation on protein N-terminal were specified as variable modifications. FDR thresholds for proteins, peptides, and modification site were specified at 1%. The minimum peptide length was set at 7. For the quantification method, TMTsixplex was selected. All other parameters in MaxQuant were set to default values. The site localization probability was set as >0.75.

### Bioinformation Analysis

#### Protein Classification

Kyoto Encyclopedia of Genes and Genomes (KEGG), protein domains, and subcellular location prediction. Gene Ontology (GO) annotation proteome was derived from the UniProt-GOA database (http://www.ebi.ac.uk/GOA/). First, the identified protein ID was converted to UniProt ID and then mapped to GO IDs by protein ID. If some identified proteins were not annotated by the UniProt-GOA database, the InterProScan soft would be used to the GO function of annotated proteins based on the protein sequence alignment method. Then, proteins were classified by GO annotation based on three categories: biological process, cellular component, and molecular function. The KEGG database was used to annotate protein pathways. InterProScan (a sequence analysis application) with the help of the protein sequence alignment method was used to annotate the functional description of identified protein domains, and the InterPro domain database was used. Wolfpsort (a subcellular localization prediction software) was selected to perform the subcellular localization analysis 51.

#### Motif Analysis

Soft motif-x52 was used to analyzes the model of sequences constituted with amino acids in specific positions of acetyl-21-mers (10 amino acids upstream and downstream of the site) in all protein sequences. Also, all database protein sequences were used as background database parameters, with other parameters in default.

#### Functional Enrichment-Based Clustering

All substrate categories which were obtained after enrichment were collated along with their *p*-values and then filtered for those categories, which were at least enriched in one of the clusters with *p* < 0.05. This filtered *p*-value matrix was transformed by the function *x* = –log_10_ (*p*-value). Finally, these *x* values were z-transformed for each category. These *z* scores were then clustered by one-way hierarchical clustering (Euclidean distance, average linkage clustering) in Genesis. Cluster membership was visualized by a heat map using the “heatmap.2” function from the “gplots” R-package.

### Protein–Protein Interaction Analysis

All differentially expressed proteins or all identified acetylated protein name identifiers were searched against the STRING database version 9.1 for protein–protein interactions. Only interactions between the proteins belonging to the searched data set were selected, thereby excluding external candidates. STRING defines a metric called “confidence score” to define interaction confidence; we fetched all interactions that had a confidence score ≥0.9 (high confidence). Interaction network from STRING was visualized in Cytoscape. A graph-theoretical clustering algorithm, molecular complex detection (MCODE), was utilized to analyze densely connected regions. MCODE is a part of the plugin tool kit of the network analysis and visualization software Cytoscape.

### Western Blot

Cells were collected, and the total protein and core histones from whole-cell lysates were extracted. About 30 μg of proteins was electrophoresed by SDS-PAGE and then transferred to nitrocellulose membranes. The proteins were probed first with primary antibodies against BATF (CST, Massachusetts, USA), IRF4 (CST), RelB (CST), RAB27A (Abcam, Cambridge, UK), H2BK12ac (PTM, Hangzhou, China), H2BK15ac (PTM), H3K9ac (PTM), H3K79ac (Active motif, Carlsbad, CA, USA), H4K77ac (PTM), and GAPDH (PTM) and then with horseradish peroxidase-coupled secondary antibodies at 1:5,000 dilution (PTM). Proteins were visualized with the antibodies using an enhanced chemiluminescence kit (GE Healthcare, Boston, MA, USA).

### Experimental Design and Statistical Method

In the present study, we used freshly isolated naive CD4^+^ T cells from the human peripheral blood and were cultured for 5 days under Tfh-polarized conditions. Naive CD4^+^ T cells and iTfh cells were harvested for quantitative analysis of global proteome and K acetylome. Reproducibility analysis of three repeated trials was evaluated by Pearson's correlation coefficient. The correlation coefficient is more than 0.75, which indicates good repeatability in this study. A *t*-test was used for statistical analysis. The fold-change cutoff was set when proteins with quantitative ratios above 1.5 or below 1/1.5 with *p* < 0.05 are deemed significant in Tfh cells compared with the naive CD4^+^ T cells group. Then, K acetylome sites were normalized by proteome proteins. The fold-change cutoff was set when proteins with quantitative ratios above 1.3 or below 1/1.3 with *p* < 0.05 are deemed significant. After performing the bioinformation analysis, such as GO and KEGG, we selected some proteins related to Tfh cell differentiation and some histones with Kac to confirm the quantitative results of MS using Western blot.

## Results

### Naive CD4^+^ T Cells Differentiate Into Tfh Cells Under Tfh-Polarizing Condition

**Figure 2:**
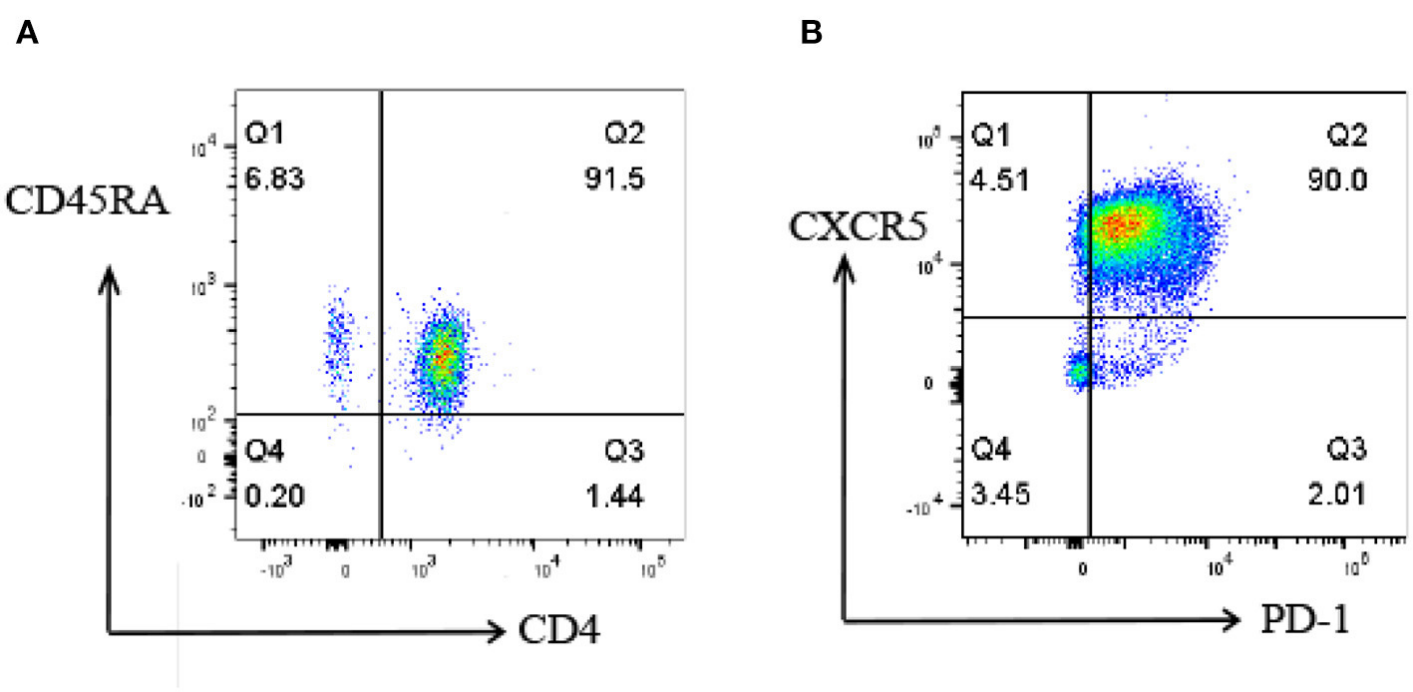
Flow cytometry analysis of naive CD4^+^ T cells and iTfh cells. **(A)** the percentage of isolated CD4^+^CD45RA^+^ T cells is 91.5%. **(B)** the percentage of CXCR5^+^PD-1^+^CD4^+^T cells is up to 90% on day 5 of *in vitro* Tfh differentiation.

To investigate the proteomic profile that was involved in the differentiation of Tfh cells, naive CD4^+^ T cells were isolated from the peripheral blood of healthy subjects (Figure 2A) and induced into Tfh cells *in vitro* under a Tfh-polarizing condition. On day 5, the cells were harvested and some marker gene expressions of Tfh cells, including CXCR5 and PD-1, were identified by flow cytometry. The results showed that the percentage of CXCR5^+^PD-1^+^CD4^+^T cells was up to 90% in the induced T cells (Figure 2B), which were consistent with the characters of Tfh cells reported in previous studies, indicating that the induction of Tfh cells was successful in this study. The iTfh cells and naive CD4^+^ T cells were used for the following proteome and K acetylome analyses.

### Comparison of Proteins Profile Between iTfh Cells and Naive CD4^+^ T Cells

Quantitative global proteome analyses were performed in triplicate on naive CD4^+^ T cells and iTfh cells using a comprehensive method, such as TMT labeling and LC-MS/MS analysis. Reproducibility analysis by Pearson's correlation coefficient confirmed the reproducibility of three repeated trials (Supplementary Figure 1). In total, 5,148 proteins were identified, among which 3,870 proteins were quantified. Among these, 802 proteins exhibited a greater than or equal to 1.5-fold increase in the expression and 598 proteins exhibited a lesser than or equal to 1.5-fold decrease in the expression in iTfh cells compared to naive CD4^+^ T cells (Supplementary Tables 1, 2).

We performed GO function classification analysis and subcellular prediction to characterize the function and subcellular location distribution of altered proteins. The number of the differentially expressed proteins in each GO term of level 2 was summed up in Supplementary Tables 3, 4. The GO distribution of upregulated and downregulated proteins includes some important biological processes, such as the metabolic process, the developmental process, and the immune system process. Moreover, according to subcellular location annotation information (Supplementary Figure 2), many more nuclear proteins were upregulated in iTfh cells, suggesting an important role of nuclear proteins in promoting the differentiation of Tfh cells.

**Figure 3:**
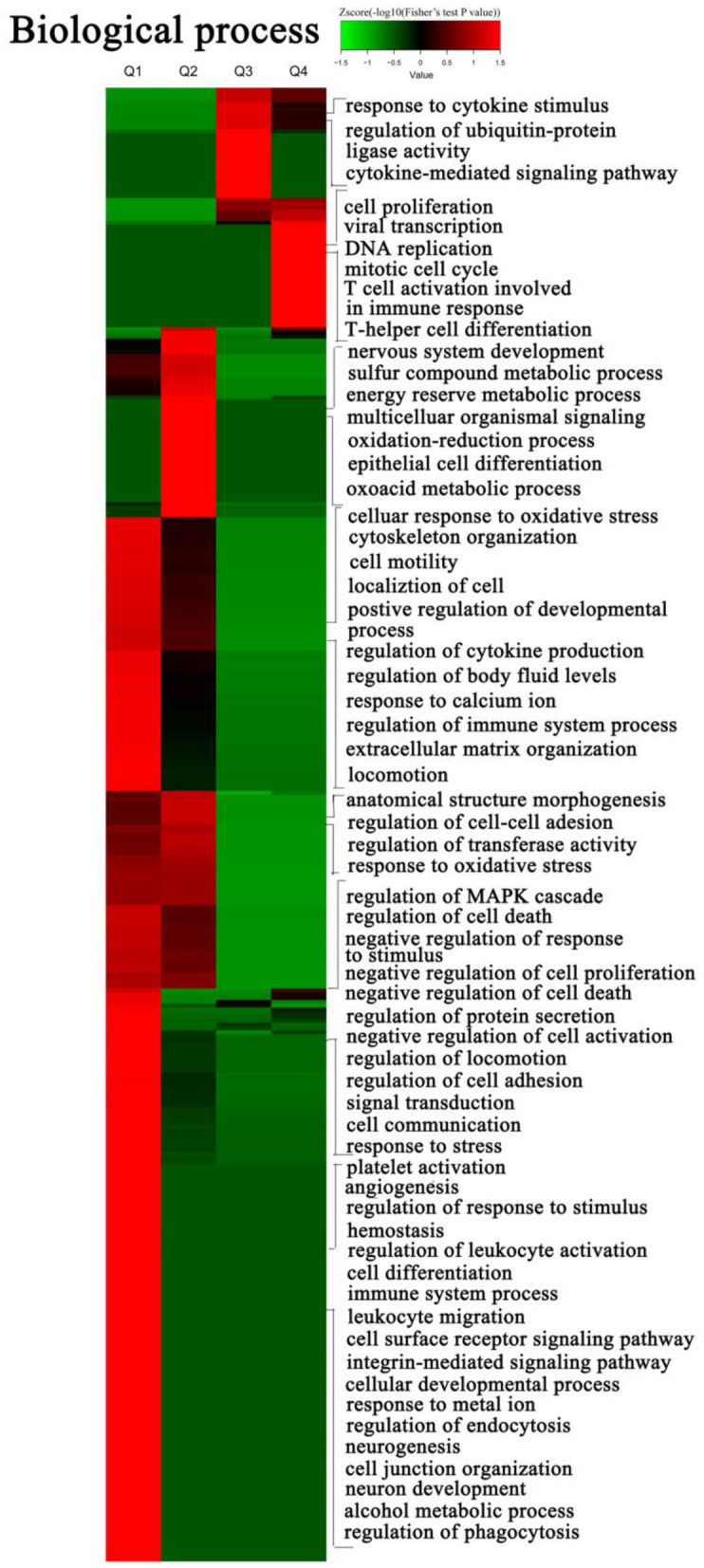
Gene ontology (GO) enrichment-based cluster analysis. The differentially expressed proteins were classified by GO annotation based on the biological process. The quantified proteins in this study were divided into four quantiles according to the quantification ratio to generate four quantiles: Q1 (0 < Ratio iTfh/Naive < 0.5), Q2 (0.5 ≤ Ratio iTfh/Naive < 0.67), Q3 (1.5 < Ratio iTfh/Naive ≤ 2), and Q4 (Ratio iTfh/Naive > 2). An enrichment analysis was performed separately in each quantile, and the overrepresented annotations were clustered through one-way hierarchical clustering for comparative analysis.

To explore the nature of the differentially expressed proteins in Tfh cell differentiation, the GO enrichment-based clustering analysis was carried out. First, the quantified proteins were divided into four quantitative categories in accordance with the quantification ratio to generate four quantitative categories: Q1 (0 < Ratio iTfh/Naive < 0.5), Q2 (0.5 ≤ Ratio iTfh/Naive < 0.67), Q3 (1.5 < Ratio iTfh/Naive ≤ 2), and Q4 (Ratio iTfh/Naive > 2). Then, we performed the clustering based on a quantitative category. The biological process was first investigated (Figure 3 and Supplementary Table 5). The upregulated proteins belong to Q4 showed the enrichments, such as DNA replication, mitotic cell cycle, viral transcription, Th cell differentiation, T cell activation, involved in the immune response and cell proliferation. Moreover, we also found some important enrichments, such as response to the stimulus of cytokines, the ubiquitin-protein ligase activity, and the cytokine-mediated signaling pathway, in the Q3 group. In the downregulated proteins in Q1, many more significant enrichments were found, including platelet activation, response to stress, regulation of response to the stimulus, and hemostasis. Interestingly, the terms of regulation of leukocyte activation and cell differentiation were also enriched in Q1, in which these proteins, such as LYN, CCL5, IL-18, and TGF-β1, have been shown to promote differentiation in other T-cell subsets. The cellular-component analysis revealed that the upregulated proteins in Q3 and Q4 were mainly located at the nuclear and that the downregulated proteins in Q1 and Q2 were mainly located at the plasma membrane (Supplementary Figure 3 and Supplementary Table 5). Moreover, we also analyzed the molecular functions of altered proteins. Nucleic acid binding was enriched in the upregulated proteins, and lipid binding and kinase activities were enriched in the downregulated proteins (Supplementary Figure 3 and Supplementary Table 5).

**Figure 4:**
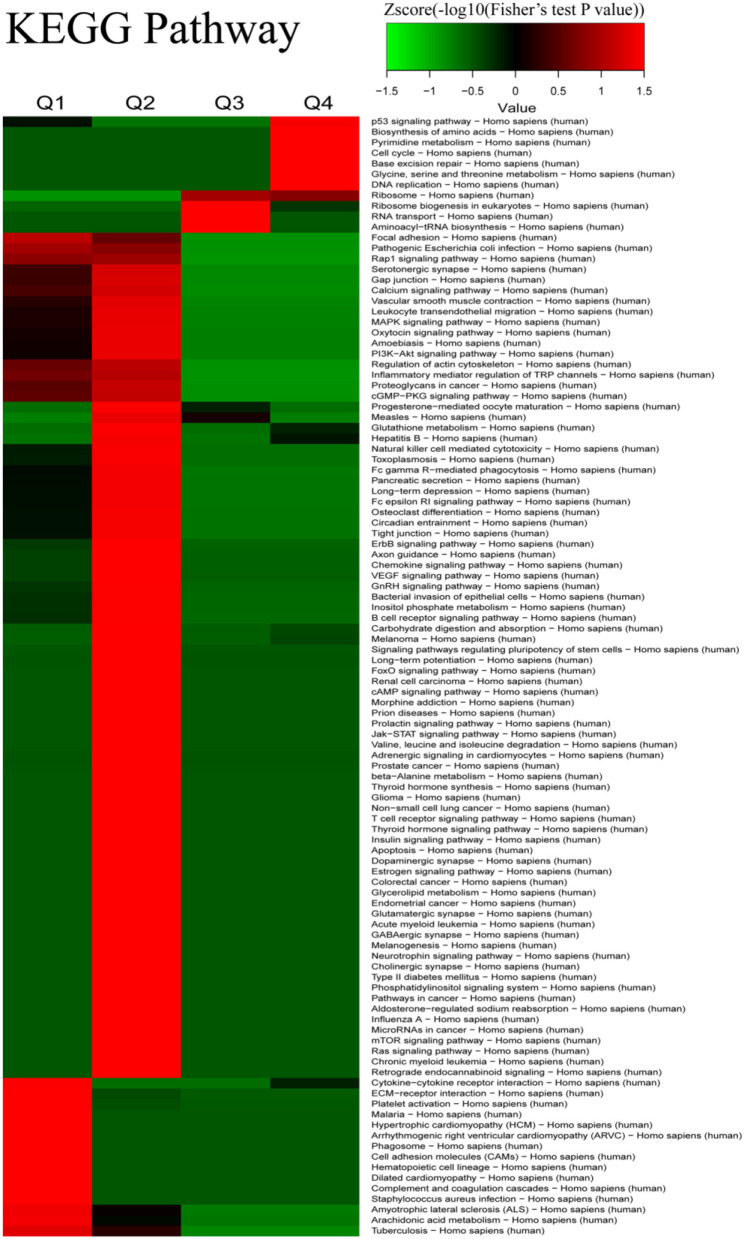
The Kyoto Encyclopedia of Genes and Genomes (KEGG) pathway-based clustering analysis for the differentially expressed proteins between iTfh cells and naive CD4^+^ T cells.

We further conducted analyses of the KEGG pathway and of the protein-complex of quantitatively altered proteins. Analyses of the KEGG pathway showed that the pathways of cytokine–cytokine receptor interaction, ECM–receptor interaction, platelet activation, focal adhesion, Rap1 signaling pathway, and pathogenic *Escherichia coli* infection were the dominant pathways enriched in the proteins with 2-fold decrease, while the proteins with 2-fold increase were involved in the p53 signaling pathway, DNA replication, cell cycle, and pyrimidine metabolism (Figure 4 and Supplementary Table 6). The results of the protein-complex analysis revealed that, among the quantitatively altered expressed proteins, Q1 was enriched with proteins involved in the ITGA2b-ITGB3 complex and Q4 was enriched with the DNA synthesome core complex, the Histone H3.1 complex, the MCM complex, and the Condensin I complex (Supplementary Figure 4 and Supplementary Table 7). In addition, we also found that the TNF-alpha/NF-kappa B signaling complex was enriched in Q3.

Following protein domain analysis (Supplementary Figure 4 and Supplementary Table 8), we detected that Q1 was enriched with proteins having SH3 domain, SH2 domain, EGF-like calcium-binding domain, EF-hand domain, integrin-β subunit, and cytoplasmic domain, whereas Q2 was enriched with proteins containing protein kinase-like domain, phosphatidylinositol 3-kinase adaptor-binding (PI3K ABD) domain, and aldehyde dehydrogenase domain. In contrast, Q3 was enriched with proteins having a zinc finger, A20-type and nucleic acid-binding, and oligosaccharide-binding (OB)-fold, while Q4 was enriched with helicase, nucleic acid-binding, DEAD/DEAH box type, OB-fold, N-terminal, C-terminal, and DNA/RNA helicase.

**Figure 5:**
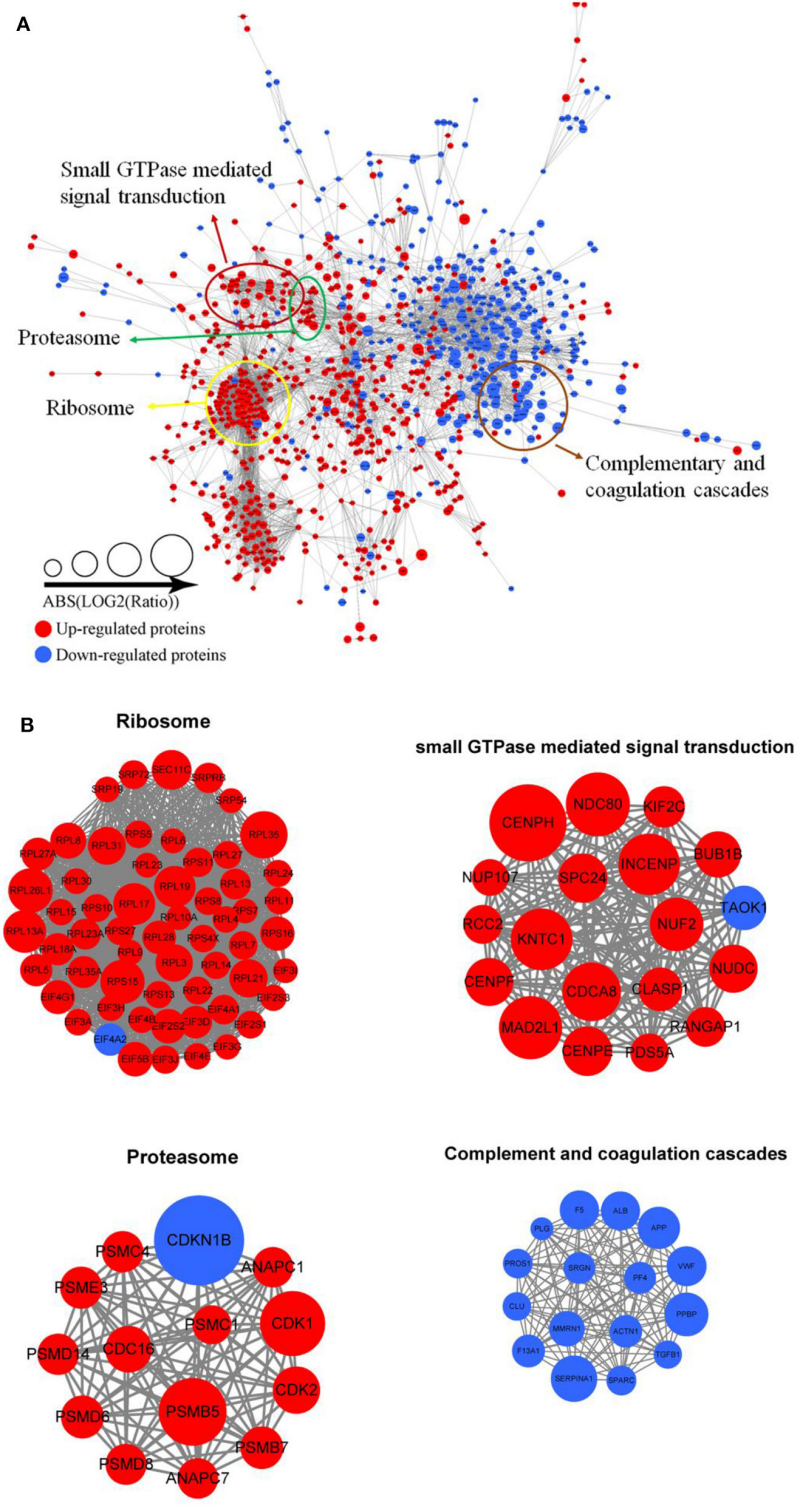
Protein–protein interaction network analyses **(A)** for all differentially expressed proteins in iTfh cells compared to naive CD4^+^ T cells. Red represents upregulated proteins and blue represents downregulated proteins. Some highly interconnected clusters of proteins were indicated **(B)**, including ribosome, Small GTPase mediated signal transduction, proteasome, and complementary and coagulation cascades.

Protein interactions are very vital to the cells, and many protein components are involved in lots of molecular processes in the cell. To investigate cellular processes during Tfh cell differentiation, we established the protein–protein interaction network in differentially expressed proteins (Figure 5A and Supplementary Table 9). We mapped differentially expressed proteins to the protein interaction database, which presents an inclusive opinion of how differentially expressed proteins accomplish different types of functions in Tfh cell differentiation. Altogether, we retrieved 35 highly interconnected clusters of proteins, including ribosome, small GTPase mediated signal transduction, proteasome, and complementary and coagulation cascades (Figure 5B).

### Alteration of K Acetylome Profile in iTfh Cells

Lysine acetylation is important in nearly all aspects of cell metabolism and is a highly conserved and reversible PTM. To investigate the dynamic changes of the whole acetylome in inducing Tfh cell differentiation, quantitative K acetylome analysis was performed using TMT labeling and Kac affinity enrichment followed by high-resolution LC-MS/MS analysis. Altogether, 281 Kac sites in 187 protein groups were recognized, among which 218 sites in 151 proteins were quantified. Then, K acetylome sites were normalized by proteome proteins, among which 173 Kac sites in 129 protein groups were covered (Supplementary Table 10). We set the fold-change cutoff to when proteins with quantitative ratios above 1.3 or below 1/1.3 and the *p* < 0.05 are deemed significant. Among the quantified proteins, 22 K acetylated proteins are upregulated and 26 K acetylated proteins are downregulated in iTfh cells compared to the naive CD4^+^ T cells (Tables 1, 2). The representative spectrum of CREBBP, XRCC5, and TCP1 proteins that underwent increased or/and decreased acetylation are presented in Supplementary Figure 5.

**Table 1:** The information of 24 upregulated lysine (K) acetylated residues corresponding to 22 proteins in iTfh cells compared to the naive CD4^+^ T cells was listed.

**Gene names**	**Position**	**Score**	**Modified sequence**	**Tfh/N ratio**	**Tfh/N *P*-value**
GNPAT	643	73.26	_LGVVEK(ac)K_	1.5838	0.0081
NPM1	229	95.502	_GQESFK(ac)K_	1.3458	0.0052
PDHB	354	89.355	_DIIFAIK(ac)K_	2.1072	0.0399
IMPDH2	134	102.07	_DVFEAK(ac)AR_	1.5019	0.0098
TCP1	400	107.06	_SLHDALCVVK(ac)R_	1.9110	0.0283
NCL	15	79.469	_NQGDPK(ac)K_	1.3205	0.0116
ME2	24	127.17	_EK(ac)GKPLMLNPR_	3.1404	0.0007
HNRNPM	48	107.17	_GEGERPAQNEK(ac)R_	1.9480	0.0033
CCT2	272	123.95	_VAEIEHAEK(ac)EK_	1.4363	0.0078
PDAP1	172	76.073	_DDATLSGK(ac)R_	1.5461	0.0248
SNX1	237	75.316	_VGKEDSSSAEFLEK(ac)R_	1.9790	0.0373
PTGES3	33	105.46	_DVNVNFEK(ac)SK_	2.2306	0.0095
CYFIP1	182	101.56	_CSVK(ac)NDHSAYK_	1.4123	0.0036
UBASH3B	208	86.898	_TEVHVEPHK(ac)K_	2.0168	0.0029
SMAP2	151	61.161	_GSEPVPEK(ac)K_	3.4721	0.0041
CREBBP	1627	84.615	_LYATMEK(ac)HK_	2.3090	0.0032
INTS4	26	114.72	_VVQPQEEIATK(ac)K_	1.8740	0.0474
NUCKS1	35	73.665	_DSGPPTK(ac)K_	2.7602	0.0326
ATPIF1	82	104.22	_HHEEEIVHHK(ac)K_	4.9739	0.0161
PSAT1	318	59.35	_GDDALEK(ac)R_	1.3576	0.0044
HIST1H1B	167	109.29	_KPAAAGVK(ac)K_	3.9791	0.0058
HIST2H2BF	12	114.7	_SAPAPK(ac)K(ac)GSK(ac)K_	1.6075	0.0048
	15	114.7	_SAPAPK(ac)K(ac)GSK(ac)K_	1.6075	0.0048
	16	91.812	_K(ac)GSK(ac)K(ac)AVTK(ac)VQK_	1.6034	0.0423

**Table 2:** The information of 32 downregulated K acetylated residues corresponding to 26 proteins in iTfh cells compared to the naive CD4^+^ T cells was listed.

**Gene names**	**Position**	**Score**	**Modified sequence**	**Tfh/N ratio**	**Tfh/N *P*-value**
ATP5H	72	116.55	_ANVAK(ac)AGLVDDFEK(ac)K_	0.5461	0.0024
PGK1	131	115.78	_FHVEEEGK(ac)GK_	0.4844	0.0053
ITGB3	234	57.59	_FNEEVK(ac)K_	0.4829	0.0025
PCCB	60	87.696	_IDAQHK(ac)R_	0.7118	0.0020
DBI	19	119.45	_TK(ac)PSDEEMLFIYGHYK_	0.3624	0.0058
LDHB	58	110.12	_SLADELALVDVLEDK(ac)LK_	0.3552	0.0007
XRCC5	565	117.38	_DQVTAQEIFQDNHEDGPTAK(ac)K_	0.3529	0.0010
FLNA	2000	109.71	_EEPCLLK(ac)R_	0.6354	0.0004
ATP5A1	239	83.862	_FNDGSDEK(ac)K_	0.7519	0.0081
MYH9	102	108.08	_VEDMAELTCLNEASVLHNLK(ac)ER_	0.3619	0.0099
TALDO1	286	176.08	_AAQASDLEK(ac)IHLDEK_	0.5140	0.0015
MDH1	103	101.65	_DLLK(ac)ANVK_	0.6556	0.0049
IDH2	280	67.032	_TDFDK(ac)NK_	0.4076	0.0130
SRP9	52	153.75	_VTDDLVCLVYK(ac)TDQAQDVK_	0.4244	0.0064
PSMB3	77	119.39	_LNLYELK(ac)EGR_	0.4425	0.0047
MECP2	22	58.079	_SEDQDLQGLK(ac)DKPLK_	0.4838	0.0022
PPIA	76	82.925	_HNGTGGK(ac)SIYGEK_	0.7223	0.0047
LMNB2	203	72.29	_K(ac)SVFEEEVR_	0.5204	0.0006
	175	66.056	_AEDGHAVAK(ac)K_	0.4718	0.0139
NNT	70	147.75	_EIFQNEK(ac)R_	0.6709	0.0032
SEPT7	208	102.87	_ADTLTPEECQQFK(ac)K_	0.5466	0.0005
UGP2	33	66.056	_QELELSVK(ac)K_	0.4858	0.0143
CREBBP	1806	146.11	_VVQHTK(ac)GCK(ac)R_	0.3912	0.0272
	1809	146.11	_VVQHTK(ac)GCK(ac)R_	0.3912	0.0272
ANKFY1	937	52.255	_VNELTK(ac)HR_	0.5852	0.0471
H2AFZ	13	97.933	_AGGK(ac)AGK(ac)DSGK(ac)AK(ac)TK_	0.4249	0.0123
	4	107.46	_AGGK(ac)AGK(ac)DSGK(ac)AK(ac)TK_	0.7581	0.0230
HIST2H3A	9	112.36	_K(ac)STGGK(ac)APR_	0.4377	0.0289
	14	112.36	_K(ac)STGGK(ac)APR_	0.4377	0.0289
	79	128.88	_EIAQDFK(ac)TDLR_	0.2308	0.0303
	18	161.67	_K(ac)QLATK(ac)AAR_	0.5686	0.0497
HIST1H4A	77	190.57	_DAVTYTEHAK(ac)R_	0.5344	0.0135

**Figure 6:**
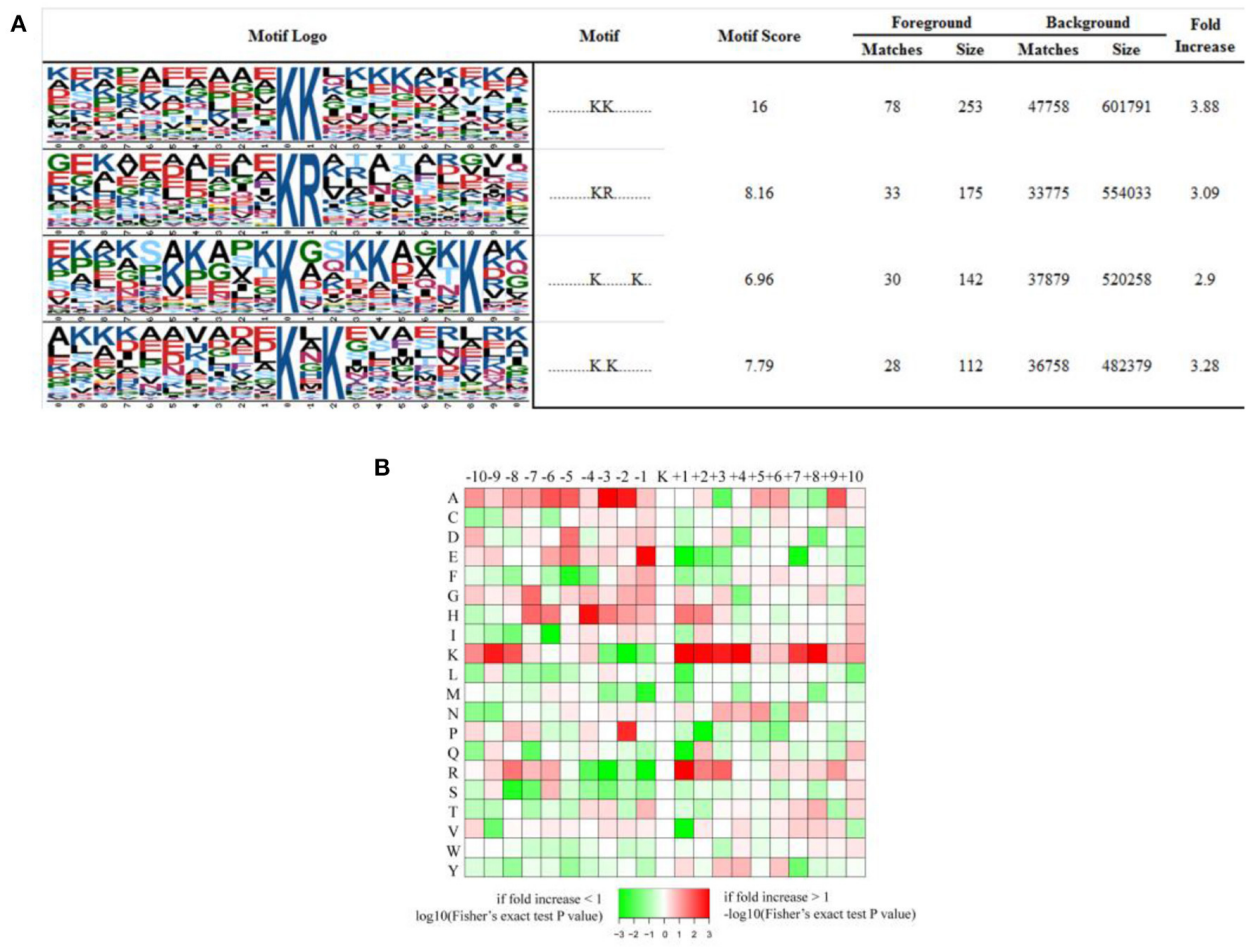
Properties of all the identified Kac peptides. **(A)** Acetylation motifs, motif score, fold increase; **(B)** Heat map of the amino acid compositions of the acetylated sites showing the frequency of the different types of amino acids around the acetylated K.

We have generated a sequence logo to calculate the probability of amino acids being over- or under-represented at the positions surrounding the acetylation site in order to identify possible specific sequence motifs around acetylated K residues in iTfh cells and naive CD4^+^ T cells. We found four significantly enriched motifs from the identified acetylated sites, namely KacK, KacR, Kac^*******^K, and Kac^*^K (Kac stands for the acetylated K and ^*^ stands for a random amino acid residue, Figure 6A). To verify whether there are specific amino acids near acetylated Ks, the amino acid sequences flanking the acetylation sites were examined by heat maps (Figure 6B). We found that two aliphatic amino acids, alanine (A) and K, occurred excessively in multiple positions surrounding Kac, indicating that they play an essential role in sequence motifs surrounding Kac. In the +1 position of Kac, K and arginine (R) were overrepresented, and glutamic acid (E) was overrepresented in the −1 position of Kac. Besides, histidine (H, −4 position) and proline (P, −2 position) were the other two amino acids whose frequencies of occurrence were comparatively high surrounding Kac.

Enrichment-based clustering analyses were performed to compare the functions of identified proteins exhibiting changed acetylation levels. The quantified acetylation proteins were divided into four quantiles (Q1–Q4) based on iTfh/N ratios for clustering analysis: Q1 (0 < Ratio T/N < 0.667), Q2 (0.667 < Ratio T/N < 0.77), Q3 (1.3 < Ratio T/N < 1.5), and Q4 (Ratio T/N > 1.5). Then, the quantifiable proteins from the four categories were plotted for GO enrichment-based cluster analysis.

**Figure 7:**
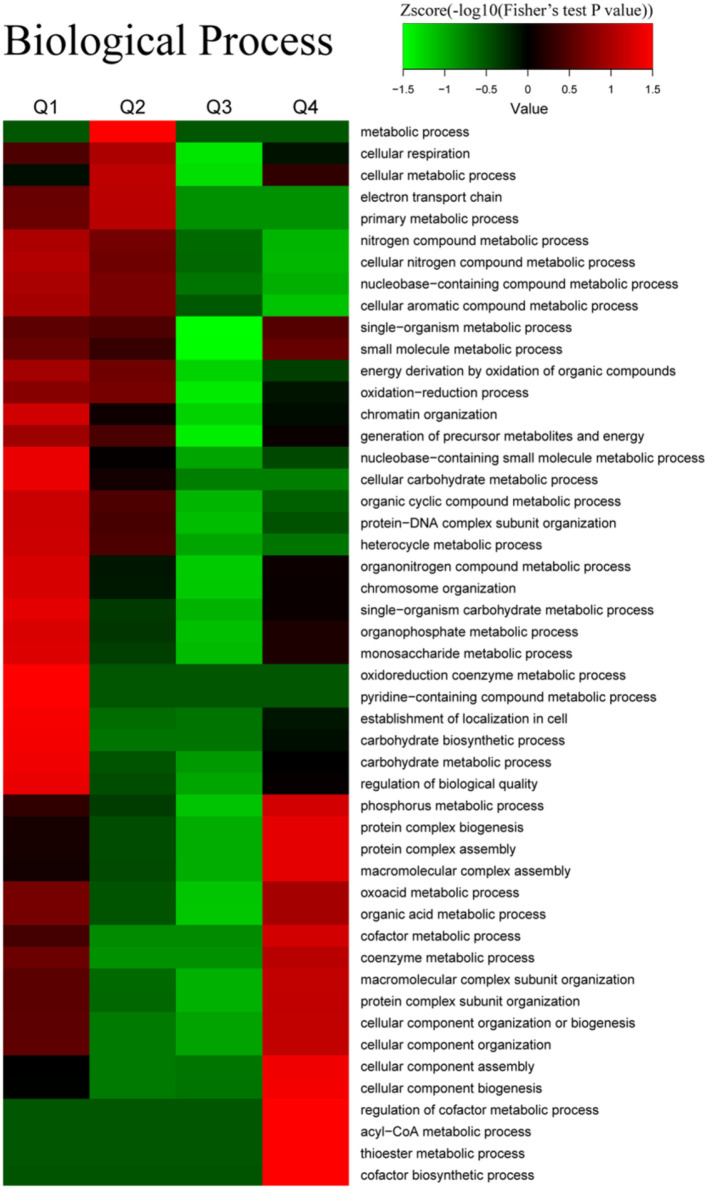
Enrichment and clustering analysis of the quantitative acetylome data sets in naive CD4^+^ T cells and iTfh cells based on the biological process. All of the quantified acetylation proteins were also divided into four quantiles (Q1–Q4) according to Tfh/N ratios: Q1 (0 < Ratio T/N < 0.667), Q2 (0.667 < Ratio T/N < 0.77), Q3 (1.3 < Ratio T/N < 1.5), and Q4 (Ratio T/N > 1.5).

The results of biological processes related to acetylation are presented in Figure 7. Decreased Kac proteins were associated with the cellular metabolic process, cellular respiration, chromatin organization, protein-DNA complex subunit organization, and the oxidation–reduction process. On the contrary, the proteins with increased Kac sites were relevant with cellular component assembly, the acyl-CoA metabolic process, the thioester metabolic process, and the cofactor metabolic process.

In the cellular component category (Supplementary Figure 6), we noticed that actin cytoskeleton, coupling factor F(o), protein complex, proton-transporting ATP synthase complex, mitochondrial membrane, DNA bending complex, and chromosomes were significantly enriched in the decreased Kac proteins. In contrast, pyruvate dehydrogenase complex, chaperonin-containing T-complex, and heterochromatin were significantly enriched in the increasing number of Kac proteins. In addition, an increase in Kac proteins was also found in the mitochondrial part and the chromosomal part.

On the ontology of molecular function (Supplementary Figure 6), coenzyme binding, coupled with the transmembrane movement of ions, ATPase activity, hydrogen ion transmembrane transporter activity, cofactor binding, rotational mechanism, and cation-transporting ATPase activity, was prominently enriched in the decline of Kac proteins. In contrast, pyruvate dehydrogenase activity, disulfide as acceptor, oxidoreductase activity, acting on the aldehyde or oxo group of donors, acetyl-CoA C-acyltransferase activity, fatty-acyl-CoA binding, and acetyltransferase activity were prominently enriched in the increase of Kac proteins.

We conducted the KEGG pathway enrichment-based clustering analysis to explore the differentially expressed Kac proteins engaged in pathways during Tfh cell differentiation (Supplementary Figure 6). Upon inducing Tfh cell differentiation, glycolysis/gluconeogenesis, citrate cycle (TCA cycle), HIF-1 signaling pathway, viral carcinogenesis, and fatty acid elongation were enriched in the upregulated Kac proteins. In contrast, 2-oxocarboxylic acid metabolism, renal cell carcinoma, alcoholism, Huntington's disease, and SLE were mainly enriched in the downregulated Kac proteins.

We found that the domains containing the NAD(P)-binding domain, histone core, lactate/malate dehydrogenase, N-terminal, and C-terminal were augmented in Q1 and Q2 proteins, exhibiting a decrease in the sites involving Kac modifications followed by protein-domain analysis. The proteins with increased Kac-modification sites in Q4 contain domains containing GroEL-like equatorial domain, GroEL-like apical domain, Aldolase-type TIM barrel, HSP20-like chaperone, and TCP-1-like chaperonin intermediate domain (Supplementary Figure 6).

### Histone Acetylation

Lysine acetylation in core histones plays a critical role in the regulation of diverse cellular processes. Kac was mainly dynamically and reversibly regulated by histone deacetylases (HDACs) and histone acetyltransferases (HATs). With three biological replicates, we identified 48 Kac sites from histones and histone isoforms, and 23 Kac sites were quantified (Supplementary Table 10). Among them, the acetylation level in four sites, namely HIST1H1B (H1K167ac), HIST2H2BF (H2BK12ac), HIST2H2BF (H2BK15ac), and HIST2H2BF (H2BK16ac), was significantly increased during Tfh cell differentiation (Table 1). In contrast, the acetylation level in seven sites, namely H2AFZ (H2AK4ac), H2AFZ (H2AK13ac), HIST2H3A (H3K9ac), HIST2H3A (H3K14ac), HIST2H3A (H3K18ac), HIST2H3A (H3K79ac), and HIST1H4A (H4K77ac), was significantly decreased during Tfh cell differentiation (Table 2). The representative spectrum of H2AK4ac, H4K77ac, and H1K167ac proteins that underwent increased or/and decreased acetylation are presented in Supplementary Figure 7.

### Validation of Proteome and Acetylome Data

**Figure 8:**
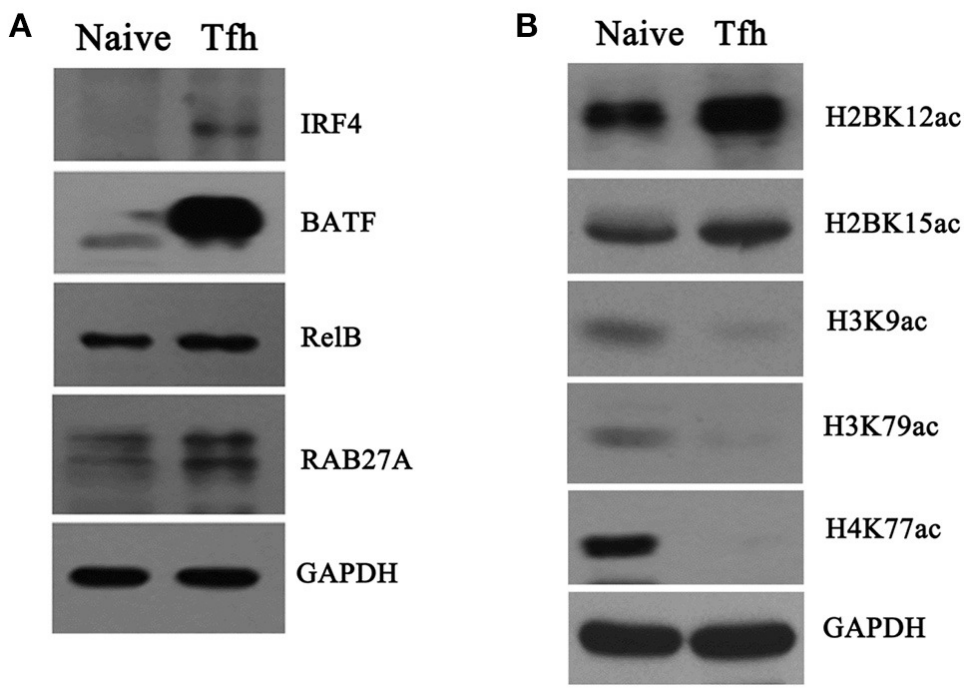
Verification of proteome and acetylome data. Naive CD4^+^ T cells were purified and induced to differentiate into Tfh cells. Naive CD4^+^ T cells and iTfh cells on day 5 were harvested, and total protein and the core histone were, respectively, extracted. **(A)** Four proteins, namely IRF4, BATF, RelB, and RAB27A, were detected by Western blot; **(B)** The acetylation of five K residues, namely H2BK12ac, H2BK15ac, H3K9ac, H3K79ac, and H4K77ac, was detected by Western blot. GAPDH was used as an inter control for protein quantification in this experiment.

To validate the differentially proteomic profiles, Western blot analysis was performed to quantify the protein levels of four upregulated genes, namely IRF4, BATF, RelB, and RAB27A, enriched in “T cell activation involved in immune response” identified by quantitative proteomics analysis. The results of Western blot showed that the protein levels of the four genes were significantly increased in iTfh cells compared with naive CD4^+^ T cells (Figure 8A), which was consistent with the results of the protein mass spectrum.

Moreover, the differentially acetylated histones were also confirmed by Western blot with sequence-specific antibodies of histone Kac. In accordance with THE quantitative results summarized in Table 2, the acetylation levels in H3K9ac, H3K79ac, and H4K77ac (Figure 8B) were significantly decreased in iTfh cells compared with naive CD4^+^ T cells. Using Western blot analysis, we also observed increased H2BK12ac and H2BK15ac levels during iTfh cells differentiation *in vitro* (Figure 8B), which was in accordance with the result greatly analyzed by MS analysis.

## Discussion

In combination with biochemistry assay, bioinformatic analysis, and quantitative proteomics, the present study revealed the quantitative proteome pattern and comprehensive Kac profile in iTfh cells and naive CD4^+^ T cells and identified the changed expression proteins and acetylated sites during Tfh cell differentiation. To date, quantitative proteomics analysis has been used to investigate proteomic changes during Th1 and Th2 cell differentiation, which revealed some important biological processes during T-cell differentiation (14, 15). Although some studies have identified that some important molecules, such as BCL6, ICOS, EBI2, LEF-1, and TCF-1, were involved in controlling Tfh cells differentiation and functions (26–29), changes to the proteomic profiles and the proteins networks during Tfh cell differentiation were still unclear. In this study, we first screened the proteomic profiles of iTfh cells and naive CD4^+^ T cells by a synthetic method using TMT labeling and LC-MS/MS analysis and identified that 802 proteins displayed a greater than or equal to 1.5-fold increased expression and 598 proteins displayed a lesser than or equal to 1.5-fold decreased expression in iTfh cells compared to naive CD4^+^ T cells. A previous study has shown different transcriptome profiles between Tfh cells and naïve CD4^+^ T cells. In the present study, we compared the proteomics data with published transcriptomics data (30). The results showed some genes with significant differences in mRNA and protein levels in Tfh cells compared with naïve CD4^+^ T cells (Supplementary Table 11), among which RelB, BATF, and RAB27A were verified by Western blot in this study. We also analyzed the possible reasons for the identification of few genes with differences in mRNA and protein levels. The transcription level of genes may be inconsistent with its protein level in cells, which are due to the translation process and PTM. In addition, because of the limitation of the technique in the method, we identified more proteins located in the cytoplasm, but we did not identify some important proteins, such as Bcl6, Tcf-1, and cMAF, in Tfh cells.

The GO term of level 2 analysis showed that the distribution of upregulated and downregulated proteins mainly includes the metabolic process, the developmental process, and the immune system process. Previous studies have shown that the engagement of a variety of specific metabolic pathways profoundly affects T-cell differentiation and function (31). Therefore, CD4^+^ Th cells differentiate into their separate functional subgroups *via* the support of distinct metabolic programs (32–34). Besides, some genes associated with T-cell activation and differentiation were included in both the upregulated gene list and the downregulated gene list. For example, IRF4, BATF, STAT3, and RELB were upregulated in iTfh cells. Recently, some findings showed that IL-21–iTfh cell differentiation of Irf4 knockout mice was impaired *in vivo*. IRF4 and STAT3 cooperatively regulate IL-21–iTfh cell differentiation (35). Store-operated Ca(2+) entry (SOCE) controlled the differentiation of Tfr and Tfh cells *via* NFAT-mediated IRF4, Bcl-6, and BATF transcription-factor expression (36). RelB belongs to the known mammalian NF-κB/Rel protein and is able to mediate transactivation directly by forming dimers with RelA and C-REL. RelB-deficient cells showed spectacular defects in IFN-gamma production and Th1 differentiation, suggesting that RelB is required for Th1 cell differentiation (37). However, whether the increased RelB expression contributes to *in vitro* differentiation of Tfh cells still needs to be investigated.

In contrast, other T subsets activation and differentiation-associated genes, such as LYN, CCL5, and TGF-β, were found in the downregulated proteins in iTfh cells. The Lyn tyrosine kinase manipulates the expression of basophil GATA-3 transcription factor and Th2 cell differentiation induction and regulates dendritic cell generation and maturation (38, 39), and its dysregulation has been linked to malignancy and autoimmunity (40, 41). CCL5 is one of the classifications of chemotactic cytokine or chemokine with a molecular weight of 8 KDa. CCL5 has a chemotactic effect on T cells, basophils, and eosinophils and has a positive effect on recruiting leukocytes into the inflammatory sites. Previous findings have shown that CCL5/CCR3 signaling promotes cellular metastasis by inducing Th2 polarization of CD4^+^ T cells (42). TGF-β has been found to induce Treg cell differentiation (43). A study found that TGF-β signaling in T cells prevented autoantibody production, self-reactive B cell activation, and Tfh cell accumulation (44). Although IL-12 and TGF-β were reported to be potent inducers for Th1, Th17, and Treg cells, IL-12 and TGF-β were added to induce human Tfh cell differentiation with IL-6 and IL-21 in the present study. Ma et al. showed that IL-12 is required for the early commitment of naive human CD4^+^ T cells to Tfh cell lineage (45). TGF-β could provide critical additional signals for STAT3 and STAT4 to promote initial Tfh cell differentiation in human (25). These previous studies have indicated that IL-12 and TGF-β may be important in the initial stage of Tfh cell differentiation.

Acetylation is a vital protein modification in cell biology, which is mainly involved in the PTM of metabolic enzymes and chromatin proteins, indicating a considerable effect on metabolism and the gene expression (46). Protein acetylation also plays a key role in the immune cells. Previous researches have shown that some key proteins, such as RORγt, IRF1, and FOXP3, were acetylated, regulating the differentiation and function of effector T cells (47–49). In the present study, the K acetylome analysis only identified limited differential acetylated proteins during Tfh cell differentiation *in vitro*, including 22 upregulated acetylated proteins and 26 downregulated acetylated proteins, when compared to naive CD4^+^ T cells, most of which were significantly enriched in the metabolic process. Interestingly, among these differentially acetylated proteins, two epigenetic regulators MeCP2 and CREBBP were downregulated in iTfh cells. Also, two proteins have been involved in regulating the immune response. Recent studies found that MeCP2 is important for the expression of sustained Foxp3 in Tregs during inflammation and was indispensable for the differentiation of naïve CD4^+^ T cells into Th1 and Th17 cells and for Th1 or Th17-mediated pathologies *in vitro* and *in vivo* (50, 51). Furthermore, MeCP2 protein undergoes multiple SUMOylation and acetylation, which impact its functions (52). MeCP2 acetylation at K464 promotes its binding to the brain derived neurotrophic factor (BDNF) promoter, and the inhibition of MeCP2 on BDNF expression was enhanced (53). P300 and SIRT1 can mediate MeCP2 acetylation and deacetylation (53). In this study, we also found an increase in the SIRT1 protein, which may be linked to the decreased acetylation of MeCP2 in iTfh cells. CREB binding protein (CREBBP) is a nuclear acetyltransferase, which can acetylate both histone and non-histone proteins to be involved in cell differentiation and growth and in regulation of the gene expression. During DNA damage response induced by the ionizing radiation, Bennetzen et al. found that nuclear acetyltransferases, EP300 and CREBBP, themselves are regulated not on the abundance level of protein but through (de)acetylation (54). They found that the four Ks (K1591, K1592, K1595, and K1597) in a K-rich cluster from position 1,591–1,597 were modified by early deacetylation (54). In this study, we found that the four Ks (K1591, K1592, K1595, and K1597) were deacetylated in iTfh cells but have no significance. In addition, we also identified some novel significantly differential acetylation in Ks of the CREBBP protein, including two downregulated Ks, K1806 and K1809, and two upregulated Ks, K1627 and K1711, in iTfh cells compared to naive T cells (Supplementary Table 10). At present, the roles of these changed Kac in CREBBP protein are still unclear. Therefore, longitudinal work is required to investigate whether acetylation of these sites regulates the activity of CREBBP, contributing to the altered histone or non-histone acetylation and Tfh cells differentiation.

Histone acetylation alters the chromatin structure and determines gene transcription (55). Naïve T cells differentiate into effector cells after being stimulated by antigens. This process is accompanied by changes in the chromatin structure and changes in acetylation of effector cytokine genes (56). The acetylation of specific histone residues analysis showed that H3(Lys-9), H4(Lys-8), and H4(Lys-12) were preferentially modified in TH1 cells, indicating that acetylation of these residues may contribute to the induction of these genes (57). Although H3 and H4 Kac were the main histones that were investigated for the gene expression regulation (58), in fact, the other two core histones, H2A and H2B, are very important in regulating the chromatin structure. Recently, Hu et al. showed that H2A.Z is highly enriched as promoters and enhancers, which is necessary for differentiation and efficient self-renewal of murine embryonic stem cells. H2A.Z deposition generates an abnormal nucleosome structure, increased chromatin accessibility, and reduced nucleosome occupancy (59). Notably, we found that H2BK12ac, H2BK15ac, and H2BK16ac were upregulated and H2AK4ac, H2AK13ac, H3K9ac, H3K14ac, H3K18ac, H3K79ac, and H4K77ac were downregulated, suggesting the increased H2B acetylation may be associated with the upregulation gene expression and the decreased H2A, H3, and H4 acetylation may be linked with transcription repression during Tfh cells differentiation *in vitro*. We speculated that H2B acetylation may control positively regulated genes for Tfh cells differentiation; in contrast, H2A, H3, and H4 acetylation may be distributed in the loci of genes preventing Tfh cell differentiation, which will be investigated by antibodies specific-chromatin immunoprecipitation (ChIP)-sequencing in the future.

## Conclusion

In conclusion, the quantitative comparison of the proteome and acetylome in primary naive CD4^+^ T cells and iTfh cells *in vitro* was studied using TMT labeling, antibody-based affinity enrichment, and high-resolution LC-MS/MS. With the help of advanced bioinformatic analysis, critical biological processes, and pathways related to T-cell activation and Tfh cell differentiation were illustrated. More importantly, histone acetylome was also identified, which may provide some potential clues for the investigation of PTMs and epigenetic mechanisms under T-cell activation and Tfh cell differentiation.

## Data Availability Statement

The mass spectrometry proteomics data have been deposited to the ProteomeXchange Consortium via the PRIDE partner repository (http://www.ebi.ac.uk/pride) with the dataset identifier PXD008563.

## Ethics Statement

The studies involving human participants were reviewed and approved by the Ethics Committee of the Second Xiangya Hospital of Central South University. The patients/participants provided their written informed consent to participate in this study.

## Author Contributions

QL and MZ designed the research. MZ, SJ, XG, HQ, RW, and HW performed research and analyzed the data. MZ, QL, and SJ wrote the paper. All authors contributed to the article and approved the submitted version.

## Conflict of Interest

The authors declare that the research was conducted in the absence of any commercial or financial relationships that could be construed as a potential conflict of interest.

## Supplementary Material

Supplementary Figure 1 Reproducibility analysis of three repeated trials of quantitative proteome analysis by Pearson's correlation coefficient.

Supplementary Figure 2 The subcellular location of upregulated **(A)** and downregulated **(B)** proteins in induced Tfh (iTfh) cells compared to naive CD4^+^ T cells.

Supplementary Figure 3 Gene ontology (GO) enrichment-based cluster analysis. The differentially expressed proteins were classified by GO annotation-based cellular compartment and molecular function. In each category, the quantified proteins in this study were divided into four quantiles according to the quantification ratio to generated four quantiles: Q1 (0 < Ratio iTfh/Naive < 0.5), Q2 (0.5 ≤ Ratio iTfh/Naive < 0.67), Q3 (1.5 < Ratio iTfh/Naive ≤ 2), and Q4 (Ratio iTfh/Naive > 2). An enrichment analysis was performed separately in each quantile for diverse categories, and the overrepresented annotations were clustered through one-way hierarchical clustering for comparative analysis.

Supplementary Figure 4 Protein complex and protein domains-based clustering analysis for the differentially expressed proteins between iTfh cells and naive CD4^+^ T cells.

Supplementary Figure 5 The representative spectrum of CREBBP **(A)**, XRCC5 **(B)**, and TCP1 **(C)** proteins that underwent increased or/and decreased acetylation in iTfh cells compared to naive CD4^+^ T cells.

Supplementary Figure 6 Enrichment and clustering analysis of the quantitative acetylome data sets in naive CD4^+^ T cells and iTfh cells based on the cellular compartment, molecular function, the KEGG pathway, and protein domains. All of the quantified acetylation proteins were also divided into four quantiles (Q1–Q4) according to Tfh/N ratios: Q1 (0 < Ratio T/N < 0.667), Q2 (0.667 < Ratio T/N < 0.77), Q3 (1.3 < Ratio T/N < 1.5), and Q4 (Ratio T/N > 1.5).

Supplementary Figure 7 The representative spectrum of H2AFZ **(A)**, HIST1H4A **(B)**, and HIST1H1B **(C)** proteins that underwent increased or/and decreased acetylation in iTfh cells compared to naive CD4^+^ T cells.

Supplementary Table 1 The upregulated proteins with more than 1.5-fold increase in iTfh cells compared to naive CD4^+^ T cells.

Supplementary Table 2 The downregulated proteins with less than 1.5-fold decrease in iTfh cells compared to naive CD4^+^ T cells.

Supplementary Table 3 GO distribution of upregulated proteins (iTfh vs. naive T cells).

Supplementary Table 4 GO distribution of downregulated proteins (iTfh vs. naive T cells).

Supplementary Table 5 GO enrichment-based cluster analysis for the differentially expressed proteins between iTfh cells and naive CD4^+^ T cells, which were divided into four quantiles according to the quantification ratio to generate four quantiles: Q1 (0 < Ratio iTfh/Naive < 0.5), Q2 (0.5 ≤ Ratio iTfh/Naive < 0.67), Q3 (1.5 < Ratio iTfh/Naive ≤ 2), and Q4 (Ratio iTfh/Naive > 2).

Supplementary Table 6 The KEGG pathway-based cluster analysis for the differentially expressed proteins between iTfh cells and naive CD4^+^ T cells.

Supplementary Table 7 Protein complex-based clustering analysis for the differentially expressed proteins between iTfh cells and naive CD4^+^ T cells.

Supplementary Table 8 Protein domain-based clustering analysis for the differentially expressed proteins between iTfh cells and naive CD4^+^ T cells.

Supplementary Table 9 Information of node and network in the Protein-protein interaction network analyses for the differentially expressed proteins between iTfh cells and naive CD4^+^ T cells.

Supplementary Table 10 The information of 24 quantified K acetylated residues in core histone in iTfh cells compared to naive CD4^+^ T cells was listed.

Supplementary Table 11 Upregulated or downregulated genes in both mRNA and protein levels between iTfh cells and naïve CD4^+^ T cells through comparing the proteomics data with published transcriptomics data.

## Abbreviations

Tfh: follicular helper T
PTM: post-translational modification
LC-MS: liquid chromatography-mass spectrometry
MS: mass spectrometry
SLE: systemic lupus erythematosus
RA: rheumatoid arthritis
DTT: dithiothreitol
KEGG: Kyoto Encyclopedia of Genes and Genomes
GO: Gene Ontology
A: Alanine
K: Lysine
Kac: Lysine acetylation
HATs: histone acetyltransferases
HDACs: histone deacetylases
ChIP: chromatin immunoprecipitation.
