# Supplementary figures and images for: Comparative Analysis of Global Proteome and Lysine Acetylome Between Naive CD4+ T Cells and CD4+ T Follicular Helper Cells
Source: Front Immunol. 2021 Mar 25;12:643441. doi: 10.3389/fimmu.2021.643441 (PMC8027069; doi:10.3389/fimmu.2021.643441)

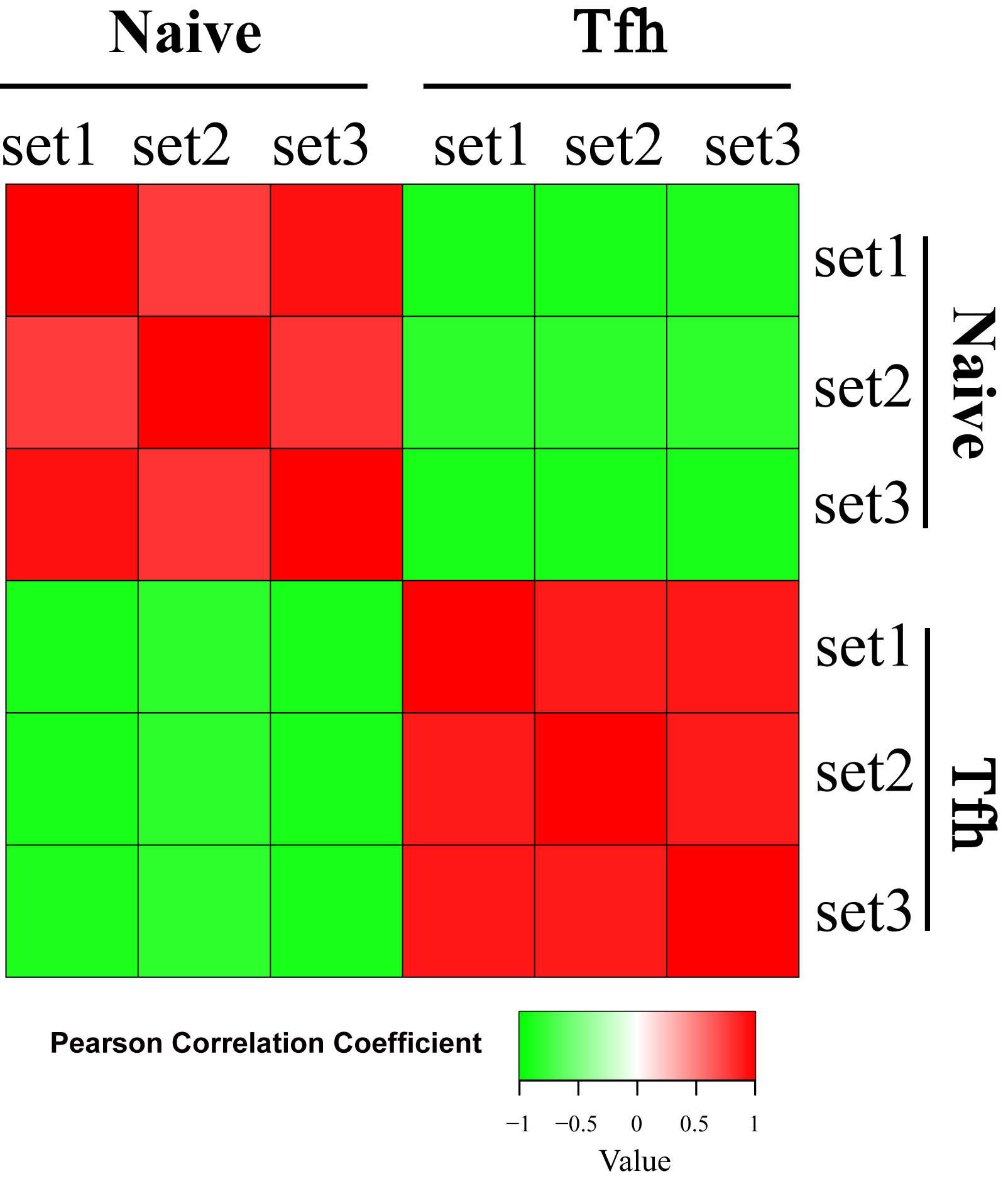

Supplement: Supplementary Figure 1 — Reproducibility analysis of three repeated trials of quantitative proteome analysis by Pearson's correlation coefficient. [file Data_Sheet_1.zip › supplementary figure S1.TIF]

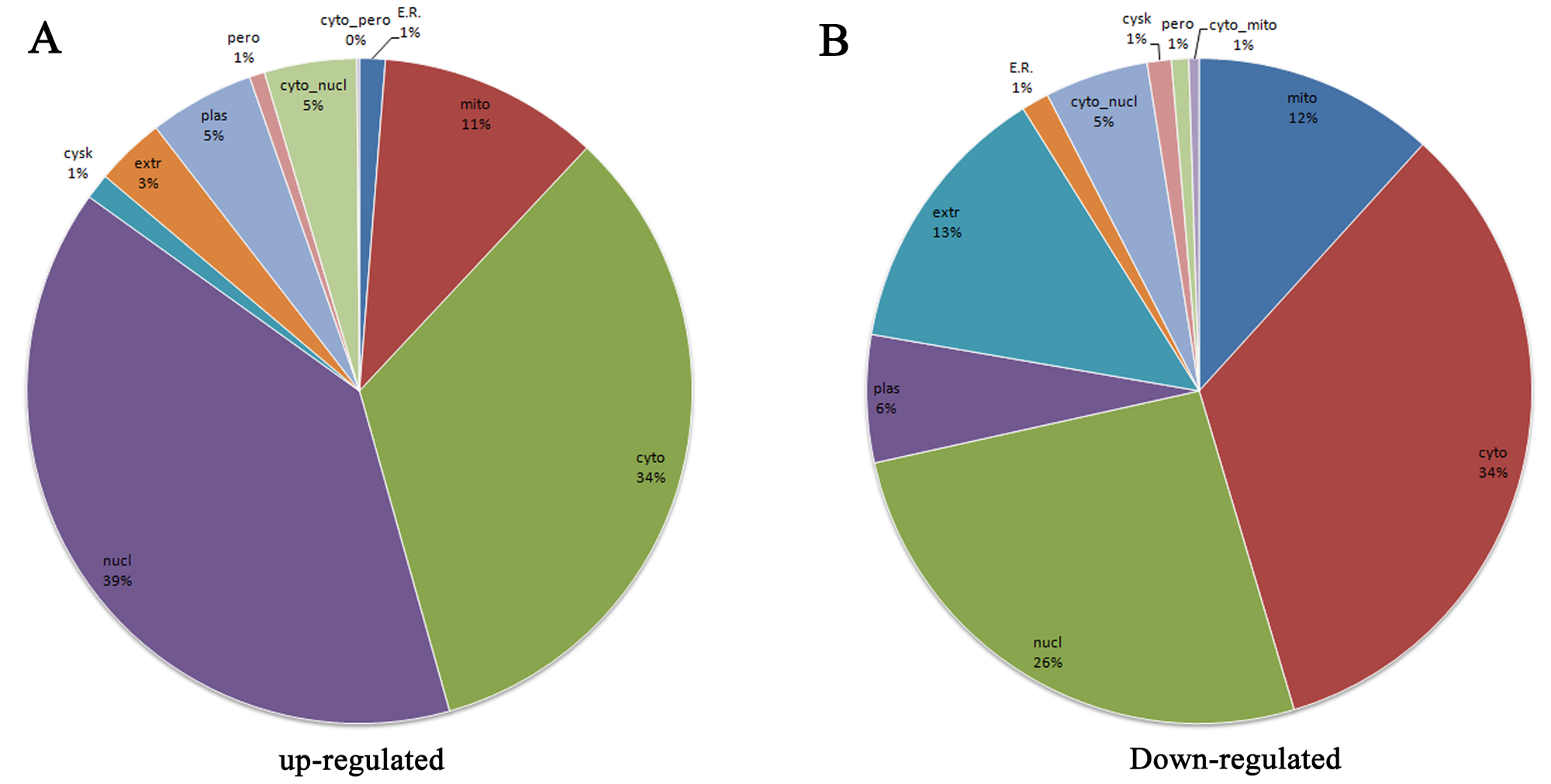

Supplement: Supplementary Figure 1 — Reproducibility analysis of three repeated trials of quantitative proteome analysis by Pearson's correlation coefficient. [file Data_Sheet_1.zip › supplementary figure S2.TIF]

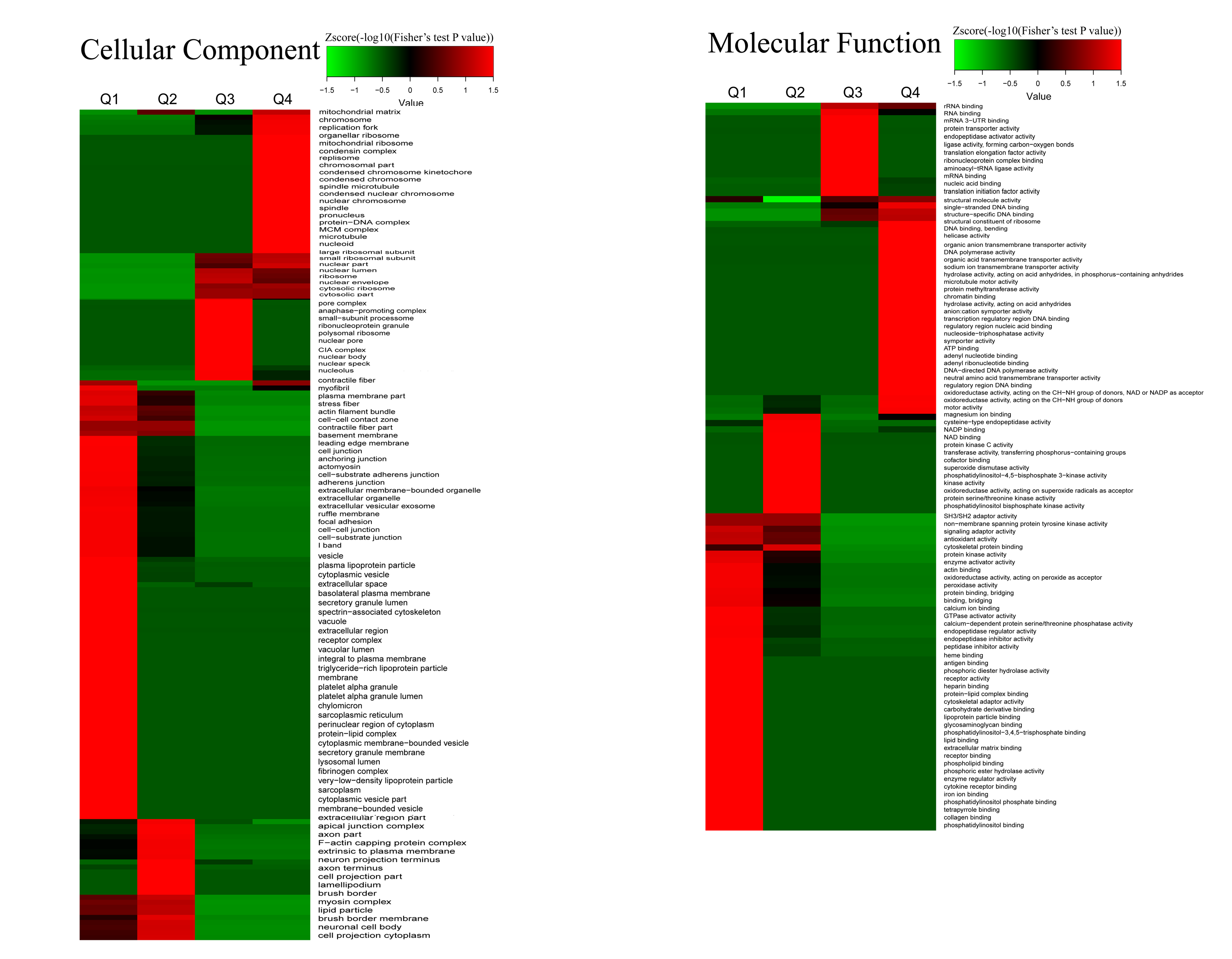

Supplement: Supplementary Figure 1 — Reproducibility analysis of three repeated trials of quantitative proteome analysis by Pearson's correlation coefficient. [file Data_Sheet_1.zip › supplementary figure S3.TIF]

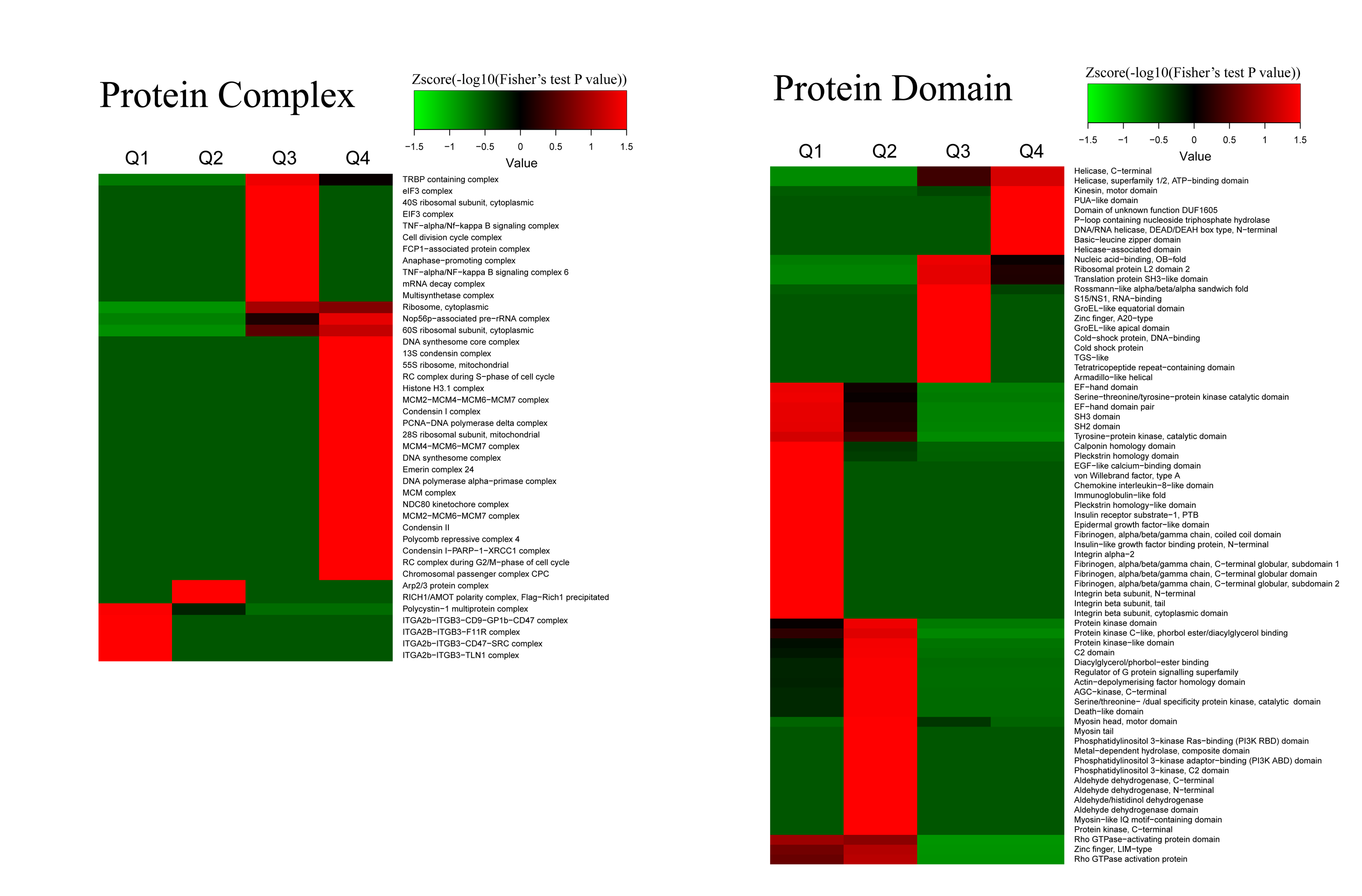

Supplement: Supplementary Figure 1 — Reproducibility analysis of three repeated trials of quantitative proteome analysis by Pearson's correlation coefficient. [file Data_Sheet_1.zip › supplementary figure S4.TIF]

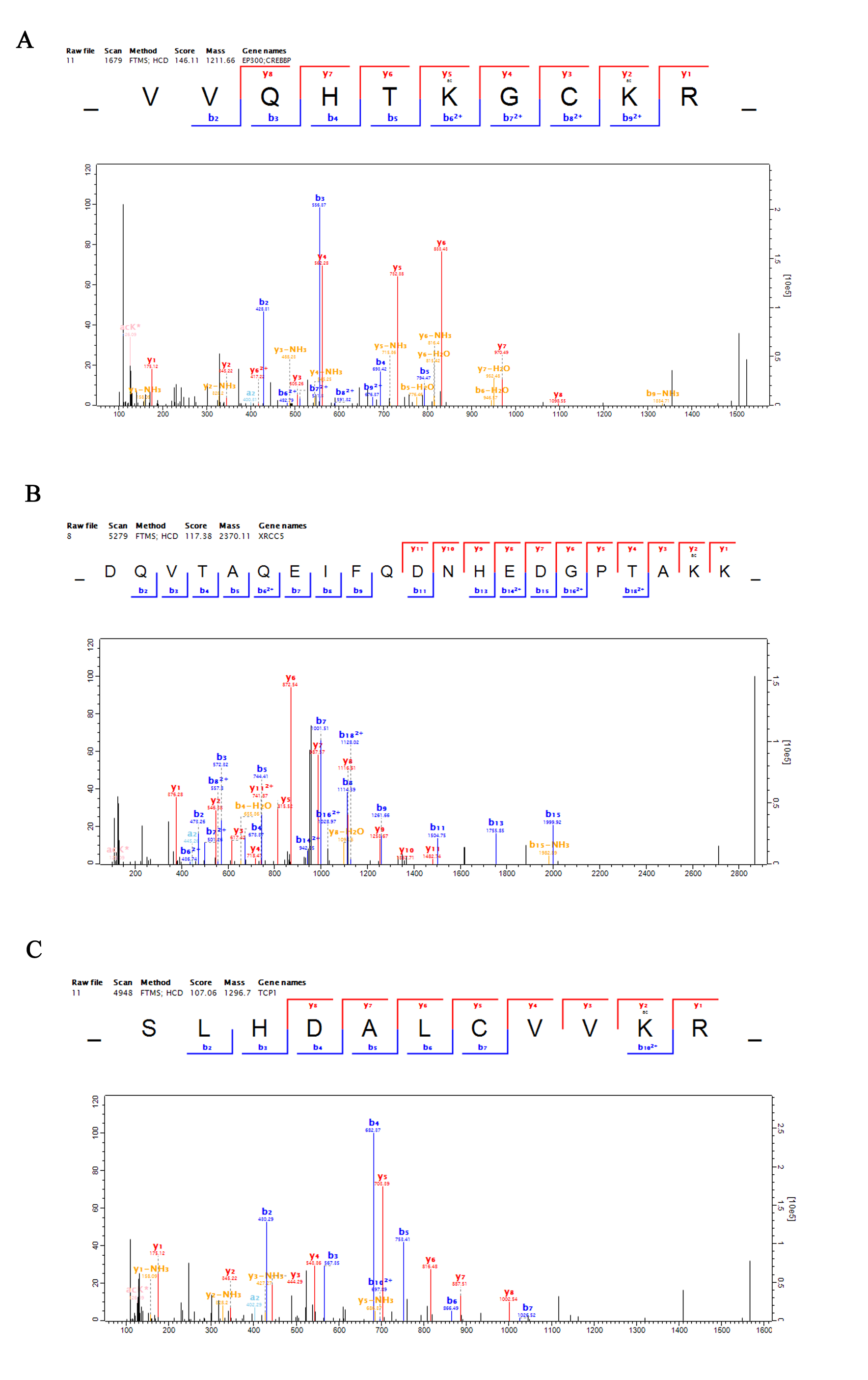

Supplement: Supplementary Figure 1 — Reproducibility analysis of three repeated trials of quantitative proteome analysis by Pearson's correlation coefficient. [file Data_Sheet_1.zip › supplementary figure S5.TIF]

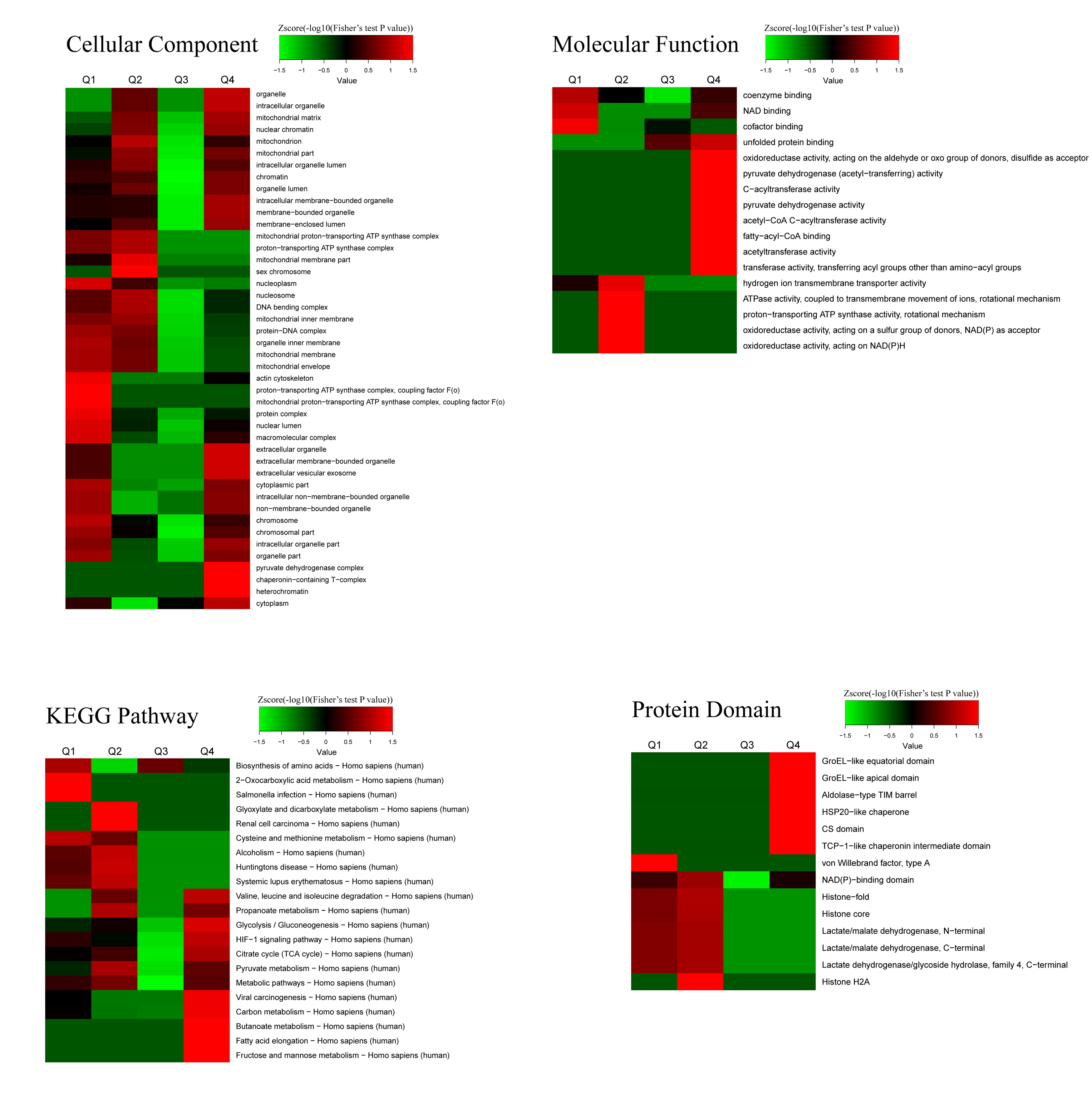

Supplement: Supplementary Figure 1 — Reproducibility analysis of three repeated trials of quantitative proteome analysis by Pearson's correlation coefficient. [file Data_Sheet_1.zip › supplementary figure S6.TIF]

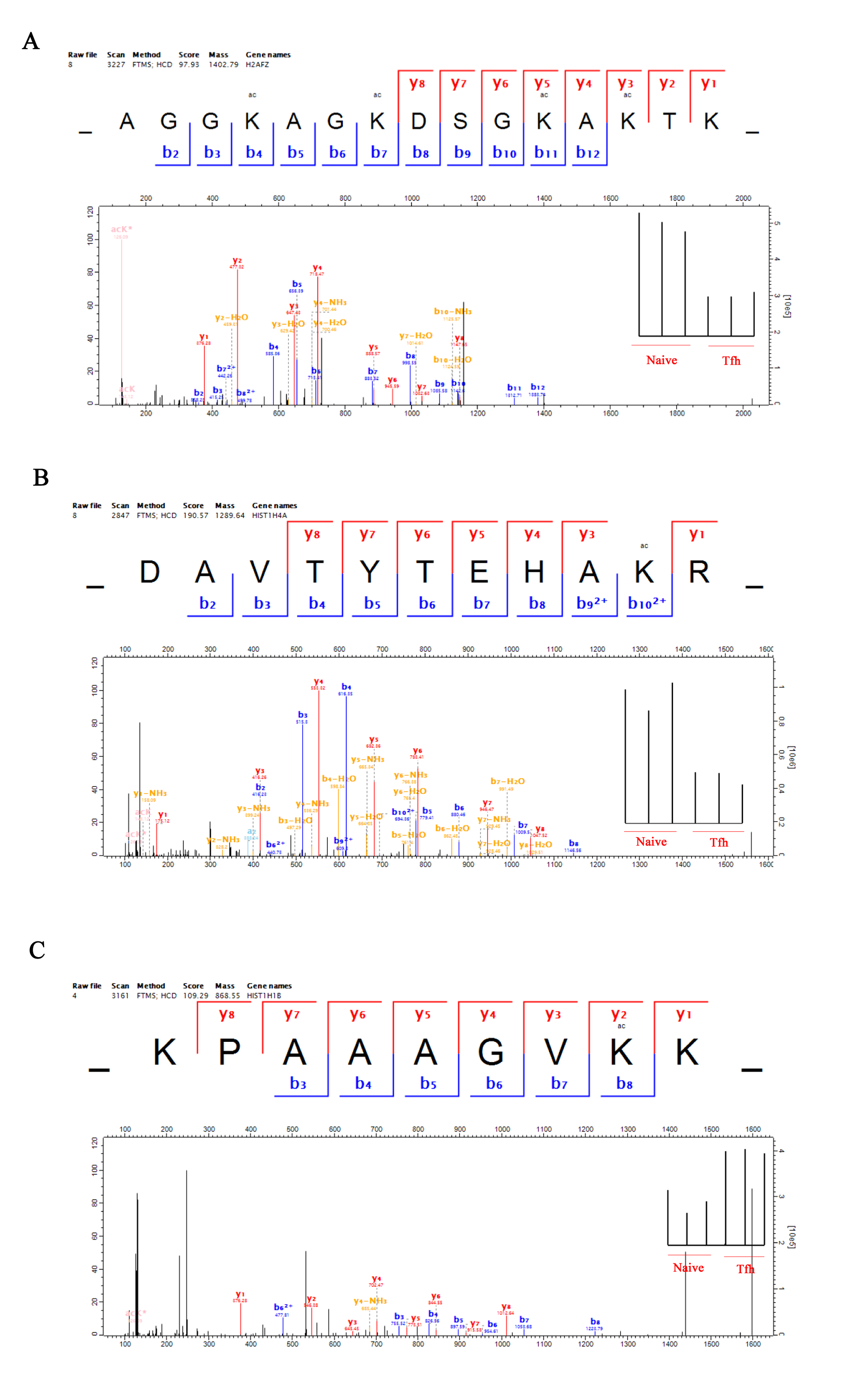

Supplement: Supplementary Figure 1 — Reproducibility analysis of three repeated trials of quantitative proteome analysis by Pearson's correlation coefficient. [file Data_Sheet_1.zip › supplementary figure S7.TIF]
